# A PRMT5-ZNF326 axis mediates innate immune activation upon replication stress

**DOI:** 10.1126/sciadv.adm9589

**Published:** 2024-06-05

**Authors:** Phuong Mai Hoang, Denis Torre, Patrick Jaynes, Jessica Ho, Kevin Mohammed, Erik Alvstad, Wan Yee Lam, Vartika Khanchandani, Jie Min Lee, Chin Min Clarissa Toh, Rui Xue Lee, Akshaya Anbuselvan, Sukchan Lee, Robert P. Sebra, Martin J. Walsh, Ivan Marazzi, Dennis Kappei, Ernesto Guccione, Anand D. Jeyasekharan

**Affiliations:** ^1^ Cancer Science Institute of Singapore, National University of Singapore, Singapore, Singapore.; ^2^ Center for OncoGenomics and Innovative Therapeutics (COGIT), Tisch Cancer Institute, Icahn School of Medicine at Mount Sinai, New York, NY 10029, USA.; ^3^ Department of Oncological Sciences, Icahn School of Medicine at Mount Sinai, New York, NY 10029, USA.; ^4^ Department of Genetic and Genomics Sciences, Icahn School of Medicine at Mount Sinai, New York, NY 10029, USA.; ^5^ Programme in Emerging Infectious Diseases, Duke-NUS Medical School, Singapore, Singapore.; ^6^ Department of Pharmacological Sciences, Icahn School of Medicine at Mount Sinai, New York, NY 10029, USA.; ^7^ Department of Biological Chemistry, University of California Irvine, Irvine, CA 92697, USA.; ^8^ Center for Epigenetics and Metabolism, University of California Irvine, Irvine, CA 92697, USA.; ^9^ Department of Integrative Biotechnology, Sungkyunkwan University, Suwon 16419, South Korea.; ^10^ Department of Biochemistry, Yong Loo Lin School of Medicine, National University of Singapore, Singapore, Singapore.; ^11^ NUS Centre for Cancer Research, Yong Loo Lin School of Medicine, National University of Singapore, Singapore, Singapore.; ^12^ Department of Haematology-Oncology, National University Hospital, Singapore, Singapore.; ^13^ Department of Medicine, Yong Loo Lin School of Medicine, National University of Singapore, Singapore, Singapore.

## Abstract

DNA replication stress (RS) is a widespread phenomenon in carcinogenesis, causing genomic instability and extensive chromatin alterations. DNA damage leads to activation of innate immune signaling, but little is known about transcriptional regulators mediating such signaling upon RS. Using a chemical screen, we identified protein arginine methyltransferase 5 (PRMT5) as a key mediator of RS-dependent induction of interferon-stimulated genes (ISGs). This response is also associated with reactivation of endogenous retroviruses (ERVs). Using quantitative mass spectrometry, we identify proteins with PRMT5-dependent symmetric dimethylarginine (SDMA) modification induced upon RS. Among these, we show that PRMT5 targets and modulates the activity of ZNF326, a zinc finger protein essential for ISG response. Our data demonstrate a role for PRMT5-mediated SDMA in the context of RS-induced transcriptional induction, affecting physiological homeostasis and cancer therapy.

## Teaser

PRMT5 promotes immune activation during replication stress.

## INTRODUCTION

Replication stress (RS) is defined as the perturbation of DNA replication, which results in stalled or collapsed replication forks (*1*). RS represents a major source of endogenous DNA damage during cell division, leading to the activation of the DNA damage response (DDR). The failure to repair such lesions affects genome integrity and has major consequences for both physiological cell homeostasis and cancer growth, as well as its potential eradication. Although RS plays a major role in malignant transformation, it is also exploited to the detriment of cancer (*2*–*4*). Examples of cancer therapeutics that induce or exacerbate RS include chemotherapeutics such as nucleoside analogs (*5*–*7*), hydroxyurea (HU) (which uncouples helicase from polymerase activity) (*8*), and inhibitors of key DDR kinases such as ataxia telangiectasia and Rad3-related protein (ATR) (*9*, *10*), checkpoint kinase 1 (CHK1), and Wee1 (*11*, *12*).

Notably, recent reports suggest that inhibiting specific DDR/RS proteins or chromatin regulators in cancer cells can trigger intrinsic immune signaling (*13*–*20*). This immune signaling cascade is initiated by pattern recognition receptors (PRRs) like RIG-I–like receptors and cytosolic nucleic acid–sensing cyclic guanosine monophosphate–adenosine monophosphate (cGAMP) synthase (cGAS), which recognize cytosolic RNA or DNA molecules, respectively. Consequently, the activation of innate immune signaling leads to the transcriptional stimulation of type I interferons (IFN-α and IFN-β) by interferon regulatory factors (IRFs) and nuclear factor κB (NF-κB), followed by the Janus kinase (JAK)/signal transducer and activator of transcription (STAT)–dependent production of cytokines and other interferon-stimulated genes (ISGs) (*21*, *22*). Cancer cells within the tumor microenvironment (TME) secrete IFNs and ISGs, which facilitate tumor antigen presentation and promote the recruitment and activation of cytotoxic T cells that drive tumor rejection (*23*).

While the cytoplasmic nucleic acid sensing molecules and signaling pathways that regulate the induction of innate immune signaling after DNA damage are well understood, less is known about chromatin regulators that influence the induction of immune signaling, particularly in the context of RS. Throughout DNA replication, several chromatin factors work in a coordinated manner to facilitate chromatin remodeling, ensure correct reestablishment of epigenetic marks, and preserve gene expression (*24*). Overall, there is limited understanding of how chromatin changes during RS are orchestrated. Here, we used HU, a well-characterized RS inducer, to identify key chromatin and transcriptional regulators acting downstream of HU. We discovered a role for PRMT5 and ZNF326 in orchestrating the innate immune response caused by RS.

We report that HU causes relocalization of PRMT5, an enzyme mainly localized in the cytoplasm, to the nucleus. This shift leads to an RS-dependent increase in nuclear SDMA signal. By mass spectrometry (MS) analysis, we demonstrate that the symmetric dimethylation of ZNF326, a component of the DBC1/ZIRD (DBIRD) transcription elongation complex (*25*), is increased and is essential in mediating the induction of RS-induced ISGs. Overall, we uncover a previously unreported role of PRMT5-mediated SDMA in the context of RS and identified pertinent SDMA proteins, which lay the foundation for future mechanistic studies on the role of PRMT5 and the RS-induced immune response.

## RESULTS

### HU activates expression of innate immune genes and ERVs

**Fig. 1.**
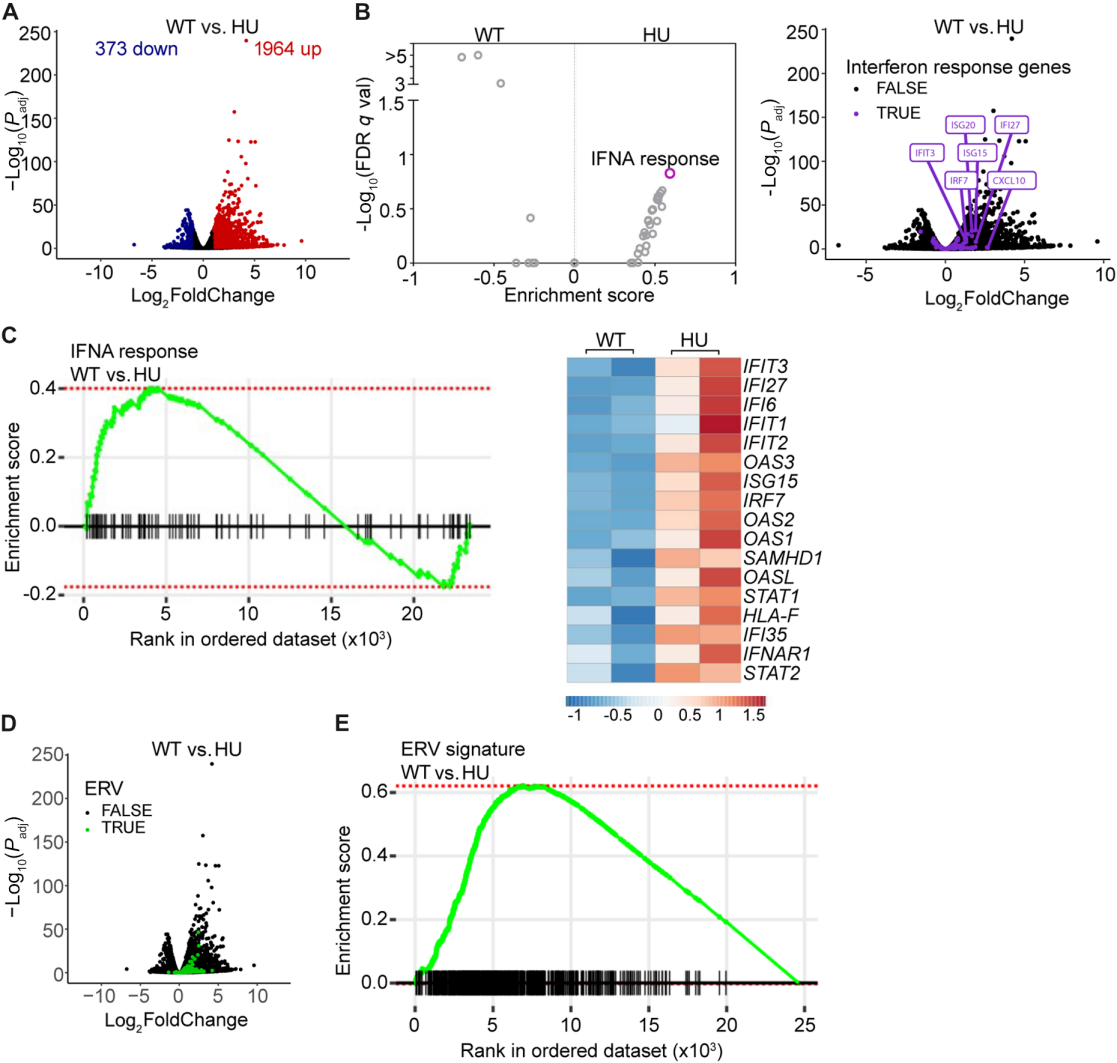
HU induces global changes to the transcriptome. (**A**) Volcano plot displaying differentially expressed genes and ERVs in MCF10A cells upon HU treatment (1 mM) for 24 hours. Up-regulated genes are defined by requiring false discovery rate (FDR) < 0.05 and log_2_FoldChange > 1 (up-regulated following HU treatment) or log_2_FoldChange < −1 (down-regulated following HU treatment). (**B**) Left: Volcano plot displaying gene set enrichment analysis (GSEA) of 50 hallmark gene sets for comparison of MCF10A ± HU (1 mM) for 24 hours. Right: Volcano plot displaying IFN response gene expression distribution in HU (1 mM) versus no treatment control for 24 hours. (**C**) Left: Enrichment plot from GSEA for comparison of MCF10A ± HU (1 mM) 24 hours. Right: Heatmap of normalized read counts from RNA-seq of MCF10A ± HU (1 mM) for 24 hours. The heatmap was generated using the ClustVis webtool (*71*). (**D**) Volcano plot displaying ERVs expression distribution in HU (1 mM) versus no treatment control for 24 hours. (**E**) Enrichment plot of ERV signature from GSEA for comparison of MCF10A treated with HU (1 mM) versus no treatment control for 24 hours.

To examine the transcriptomic changes caused by RS, we performed RNA sequencing (RNA-seq) in a nontransformed breast epithelial cell line (MCF10A) treated with HU for 24 hours. Twenty-four hours of exposure to HU were sufficient to induce DDRs and cell cycle arrest in MCF10A cells (fig. S1). HU treatment significantly altered expression of multiple genes, with 1964 being up-regulated and 373 down-regulated (Fig. 1A and table S1). Gene set enrichment analysis (GSEA) for hallmark gene sets representing well-defined biological pathways revealed various signatures affected by HU treatment (table S1). The gene set associated with IFN-α (IFNA) response hallmark was the most substantially enriched (Fig. 1B), and HU strongly induced ISGs such as interferon-induced proteins with tetratricopeptide repeats (IFITs), ISG15, and IRF7 (Fig. 1C). As a source of cytosolic nucleic acids that affect innate immune signaling, we also focused our attention on repetitive elements. Endogenous retroviruses (ERVs) are increasingly recognized for their role in immune response activation after DNA damage (*26*). We sought to investigate if ERVs associate with HU-induced transcriptomic changes in innate immune signaling. HU strongly induced ERVs expression (Fig. 1D). This finding was corroborated in a GSEA of our constructed ERV signature gene set (Fig. 1E). Overall, these data point to up-regulation of ISGs being a prominent transcriptional response to RS, with reexpression of ERVs being a possible contributing factor.

### A chemical genetic screen identifies key epigenetic modulators of HU-induced transcriptional changes

**Fig. 2.**
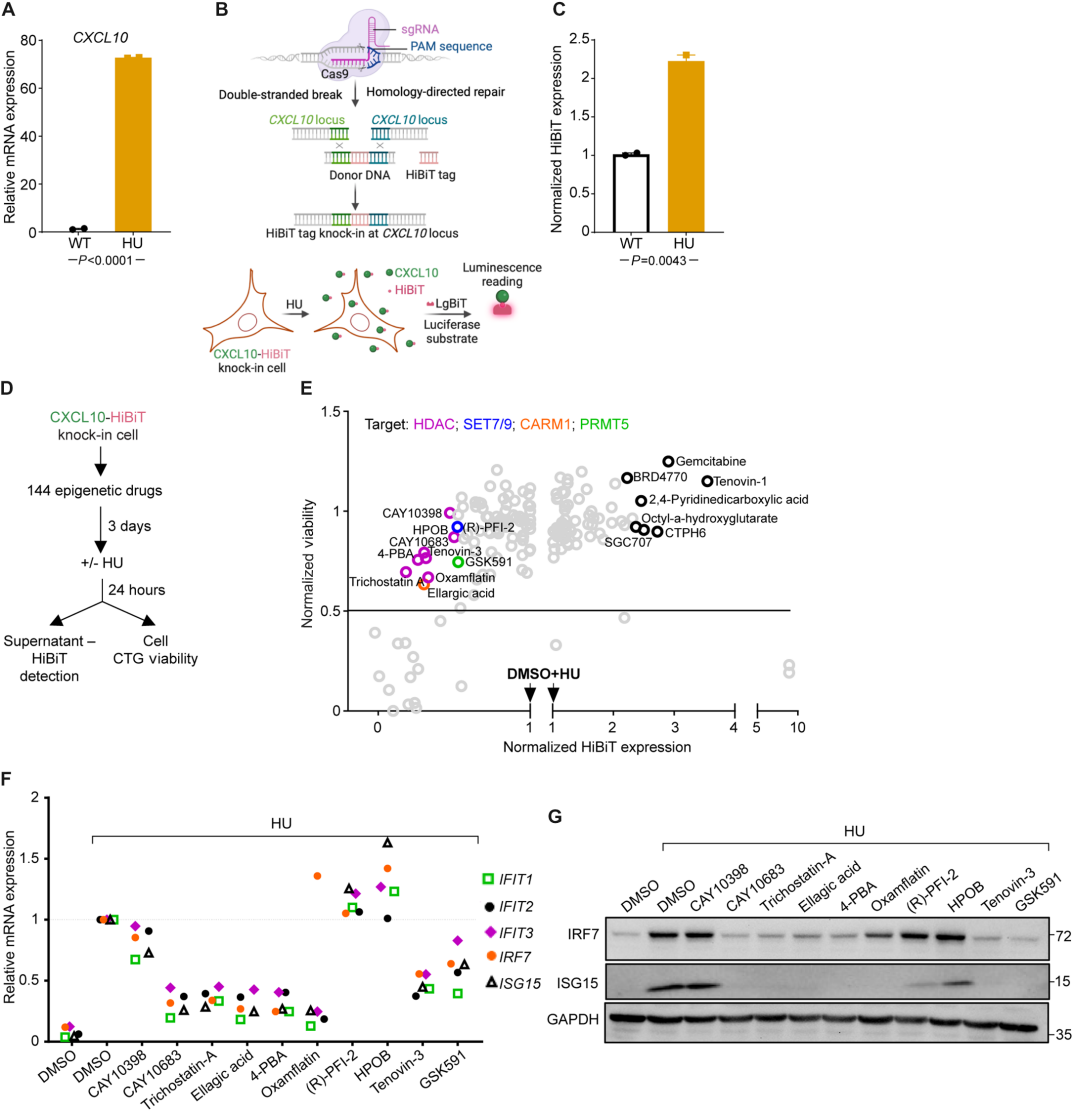
A chemical genetic screen identifies key epigenetic modulators of HU-induced transcriptional changes. (**A**) qPCR of *CXCL10* expression from MCF10A untreated or treated with HU (1 mM). Statistical significance was analyzed by unpaired Student’s *t* test of values obtained from two independent experiments. (**B**) Schematic workflow for generating HiBiT tag knock-in to *CXCL10* locus in MCF10A and HiBiT-CXCL10 detection by luminescence. The illustration was created with BioRender. (**C**) Luminescence readout from HiBiT-CXCL10 knock-in cell clone ± HU for 24 hours. (**D**) Workflow for epigenetic drug screen to determine modulators of HU-induced CXCL10 expression. (**E**) Scatterplot for epigenetic drugs down-/up-regulated HU-induced CXCL10 expression. (**F** and **G**) Validated hits from (E) with cotreated HU and epigenetic drugs for 24 hours in MCF10A WT cell by qPCR (F) and Western blot (G).

We conducted a chemical drug screen with a library of epigenetic inhibitors to pinpoint key chromatin and transcriptional regulators downstream of RS induced by HU. As a readout for the screen, we selected CXCL10, a chemokine whose expression was strongly up-regulated upon HU treatment (Fig. 2A). For high-throughput estimation of CXCL10, we knocked in a HiBiT tag into the *CXCL10* locus to aid the detection of endogenously expressed CXCL10 by a luminescent signal (*27*) (Fig. 2B). HiBiT-tagged CXCL10-expressing MCF10A single-cell clones were confirmed via Sanger sequencing, and HU treatment of these cells showed robust increases in luminescence (Fig. 2C).

For the chemical screen, the HiBiT knock-in clone was pretreated with a library of epigenetic drugs and subsequently exposed to HU for 24 hours (Fig. 2D). To eliminate drugs that decrease CXCL10 expression due to cytotoxicity, a 70% cell viability threshold was implemented. Epigenetic inhibitor compounds targeting histone deacetylases (HDACs), lysine methyltransferase (SET7/9), coactivator-associated arginine methyltransferase 1 (CARM1/PRMT4), and protein arginine methyltransferase 5 (PRMT5) emerged as the top 10 hits that negatively regulated HiBiT-CXCL10 expression (Fig. 2E). For validation, wild-type (WT) MCF10A cells were cotreated with HU and the respective epigenetic inhibitor hits and the resulting expression of ISGs was evaluated. With the exception of (R)-PFI-2 (SET7/9 inhibitor) and CAY10398 and HPOB (HDAC inhibitors), the remaining seven compounds successfully suppressed ISG expression at both mRNA and protein levels (Fig. 2, F and G). While previous studies have investigated the contributions of HDAC inhibitors (*28*–*30*) and ellagic acid (CARM1 inhibitor) (*31*) to immune response regulation, the role of PRMT5 in this phenomenon was understudied. We therefore set out to elucidate the mechanism underlying PRMT5-mediated regulation of ISG expression.

### PRMT5 regulates HU-induced ISG and ERV expression

**Fig. 3.**
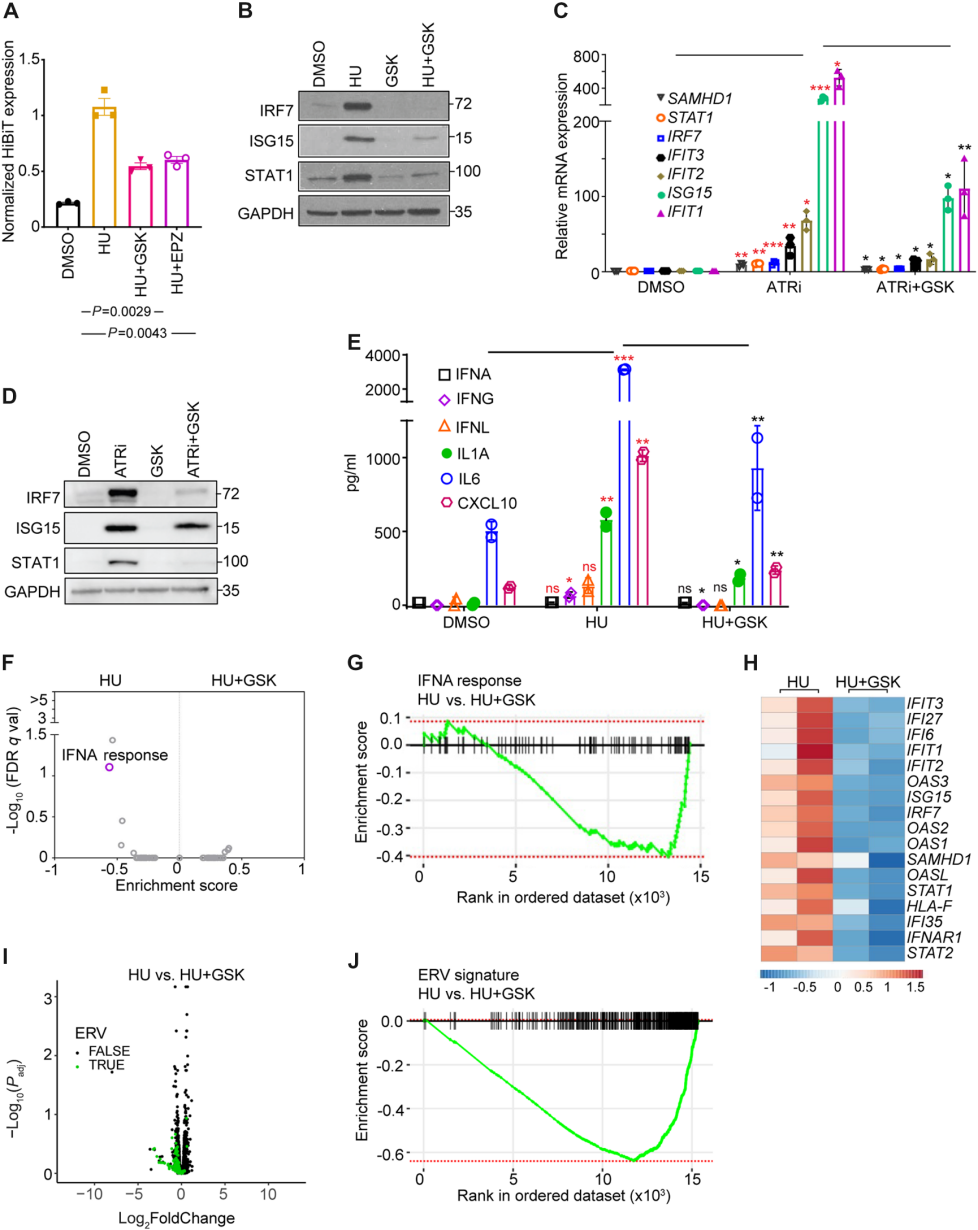
RS induces a PRMT5-dependent ISG expression pattern. (**A**) CXCL10-HiBiT expression from CXCL10-HiBiT knock-in MCF10A cells treated with DMSO, HU (1 mM), HU (1 mM) + GSK591 (1 μM), and HU (1 mM) + EPZ015666 (1 μM) for 48 hours. Statistical significance was analyzed by unpaired Student’s *t* test of values obtained from three independent experiments. (**B**) Western blot of MCF10A treated with DMSO, HU, GSK591, and HU + GSK591 and blot for IRF7, ISG15, STAT1, and GAPDH (glyceraldehyde-3-phosphate dehydrogenase). (**C**) qPCR of MCF10A cells treated with DMSO, AZD6738 (ATRi) (5 μM), and ATRi (5 μM) + GSK591 (1 μM) for 6 days. Statistical significance was analyzed by unpaired Student’s *t* test of values from three independent experiments (*P ≤ 0.05; **P ≤ 0.01; ***P ≤ 0.001). Red asterisk, statistical difference between DMSO versus ATRi; black asterisk, statistical difference between ATRi versus ATRi + GSK591. (**D**) Western blot of MCF10A treated as in (C). (**E**) Multiplex ELISA for IFNs and cytokines from supernatants of MCF10A treated as in (B). Red asterisk, statistical difference between DMSO versus HU; black asterisk, statistical difference between HU versus HU + GSK591. *P* value was analyzed by unpaired Student’s *t* test of values from two independent experiments (*P ≤ 0.05; **P ≤ 0.01; ***P ≤ 0.001; ns, not significant). (**F**) Volcano plot displaying GSEA of 50 hallmark gene sets comparing MCF10A treated with HU versus HU + GSK591 for 24 hours. (**G**) Enrichment plot of GSEA IFNA hallmark comparing MCF10A treated with HU versus HU + GSK591 for 24 hours. (**H**) Heatmap of normalized read counts from RNA-seq of MCF10A treated with HU versus HU + GSK591 for 24 hours. The heatmap was generated using the ClustVis webtool (*71*). (**I**) Volcano plot displaying ERV expression distribution in MCF10A treated with HU versus HU + GSK591 for 24 hours. (**J**) Enrichment plot of ERV signature from GSEA comparing MCF10A treated with HU versus HU + GSK591 for 24 hours.

To ensure that the observed PRMT5 inhibitor-associated attenuation of ISG expression downstream of RS was not specific to a single inhibitor (i.e., GSK591), we confirmed our findings with an alternative PRMT5 inhibitor, EPZ015666. CXCL10 expression in HU-treated HiBiT-CXCL10 MCF10A cells was reduced when cotreated with either EPZ015666 or GSK591 (Fig. 3A). We also confirmed that addition of GSK591 diminished the HU-induced increase in IRF7, ISG15, and STAT1 protein expression (Fig. 3B). To rule out the possibility of a cell line–specific phenotype, we evaluated and observed similar effects in a gastric cancer cell line, MKN7 (fig. S2A). To rule out that PRMT5-dependent up-regulation of ISG expression could be a HU-specific phenomenon, we used another clinical-grade RS inducer, the ATR inhibitor AZD6738. Similar enhancement of ISG expression upon ATR inhibition, as well as consequent attenuation of ISG expression with PRMT5 inhibition were observed with AZD6738 (Fig. 3, C and D). This demonstrates that PRMT5-related modulation of ISG expression is likely to be an RS generalized phenomenon.

IFNs, released among other instances during DNA damage (*20*), are initiators of the JAK-STAT signaling pathway that drives ISG transcription (*21*). We therefore evaluated the effect of PRMT5 on IFNs expression. We conducted a multiplexed enzyme-linked immunosorbent assay (ELISA) to detect the levels of IFNs and cytokines in the supernatant of dimethyl sulfoxide (DMSO), HU only, and HU + GSK591–treated MCF10A cells. PRMT5 inhibition (GSK591) diminished levels of CXCL10 and interleukin-1α (IL-1α) in the supernatant of HU-treated MCF10A cells. However, IFN levels remained relatively unperturbed (Fig. 3E and fig. S2B) except for a PRMT5-dependent transcriptional increase of IFN-λ (fig. S2C). It is known that different DNA damage agents are able to induce IFNs (*20*); therefore, we investigated whether the observed induction of IFN-stimulated genes was due to RS or double-strand break formation by treating MCF10A cells with etoposide or HU and checked for expression of IFN response after short period (8 hours) or longer period (24 hours). We observed that at both 8- and 24-hour time points, etoposide caused double-strand breaks as indicated by elevated γH2AX, but the expression of IFN-stimulated genes was similar to DMSO-treated cells. On the contrary, 24-hour exposure to HU induced IFN-stimulated genes in a PRMT5-dependent manner (fig. S2D). We were also interested in understanding the broader PRMT5-dependent transcriptional alterations after HU treatment and performed RNA-seq on HU-treated and HU + GSK591–treated cells. As expected, PRMT5 inhibition dampened the HU-induced IFN-α response (Fig. 3, F and G) with reduction in transcription of ISGs (Fig. 3H) and ERVs (Fig. 3, I and J).

Both HU treatment and PRMT5 deficiency have been independently reported to affect cell cycle progression, DDR, cell viability, and splicing (*32*). Therefore, we investigated the aforementioned phenotypes in the context of HU and GSK591 cotreatment. PRMT5 inhibition, at the tested time points, did not affect cell viability (fig. S3A). Furthermore, while HU treatment caused an S-phase cell cycle arrest in 80% of the treated cell population, addition of GSK591 did not further alter the cell cycle profile (fig. S3B). Both HU-treated and HU + GSK591–treated cells had overcome S-phase cell cycle arrest 6 days after treatment but were eventually arrested in G_2_ phase. PRMT5 inhibition resulted in only ~5% more cells to be arrested in S phase (fig. S3C). Moreover, PRMT5 inhibition did not result in further increments of HU-induced γH2AX expression and Rad51 foci formation (fig. S3, D and E). Hence, PRMT5 is unlikely to have a crucial role in influencing cell viability, cell cycle progression, and DDR signaling in the early phase (~24 hours) after induction of RS.

Lastly, while prolonged PRMT5 inhibition is known to cause widespread intron retention and exon skipping (*33*), we did not observe major changes to splicing upon short-term (24 hours) PRMT5 inhibition (fig. S3, F and G). Thus, while PRMT5 is nonessential for early HU-induced DDR signaling, cell cycle arrest, cytotoxicity, and alternative splicing, it appears to be critical for HU-induced ISG expression.

### HU triggers PRMT5 nuclear translocation and increases nuclear SDMA

**Fig. 4.**
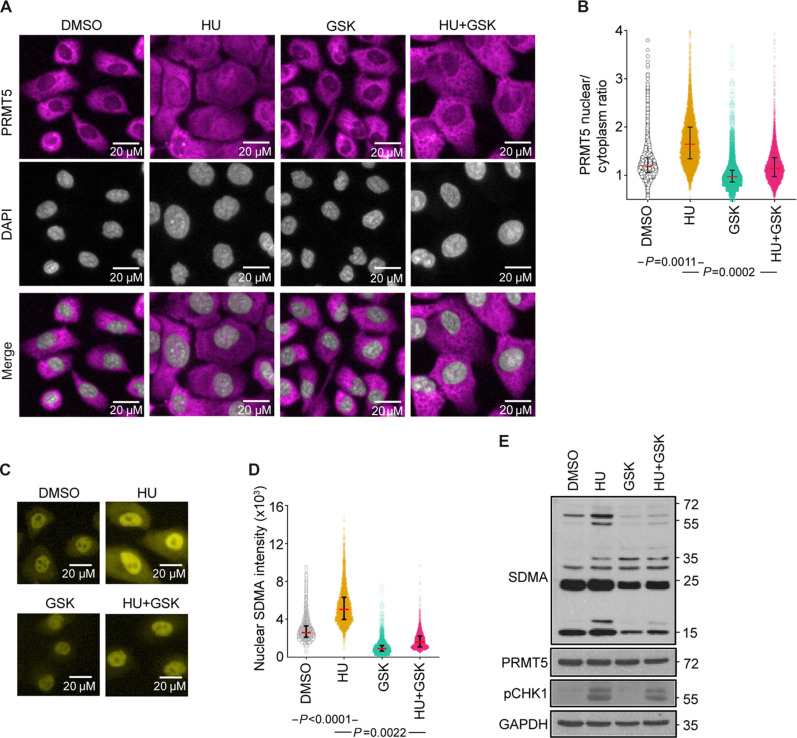
HU leads to an increase in nuclear SDMA. (**A**) MCF10A cells treated with DMSO, HU (1 mM), GSK591(1 μM), and HU (1 mM) + GSK591 (1 μM) for 24 hours and stained for PRMT5. Images were captured and analyzed using the Operetta system. (**B**) A violin plot displaying median value with interquartile range for the ratio for nuclear PRMT5 staining intensity over cytoplasmic PRMT5 staining intensity from at least 1000 cells from (A). *P* value was analyzed by unpaired Student’s *t* test of median values obtained from three independent experiments. (**C**) MCF10A cells treated with DMSO, HU, GSK591, and HU + GSK591 and stained for SDMA. Images were captured and analyzed using the Operetta system. Representative images in one of three independent experiment are shown. (**D**) A violin plot displaying median value with interquartile range for the nuclear SDMA staining intensity from at least 1000 cells from (C). *P* value was analyzed by unpaired Student’s *t* test of median values obtained from three independent experiments. (**E**) Western blot for MCF10A cells treated as in (C).

To understand alterations in PRMT5 activity upon HU treatment, we tracked, via immunofluorescence and Western blot analysis of nuclear and cytoplasm fractions, the intracellular localization of PRMT5 (fig. S4A). We noticed that HU treatment induced the translocation of PRMT5 from the cytoplasm to the nucleus. However, upon combination treatment with HU and GSK591, a reversion of PRMT5 from the nucleus back to the cytoplasm was observed (Fig. 4A). We quantitatively demonstrate this phenomenon through a per-cell PRMT5 nuclear/cytoplasm ratio (dividing nuclear PRMT5 intensity over cytoplasmic PRMT5 intensity). Consistent with the staining pattern, PRMT5 nuclear/cytoplasm ratio was higher in HU-treated cells compared to DMSO and declined upon addition of GSK591 (Fig. 4B).

As HU-treated cells are arrested in the S phase of the cell cycle, we checked whether the nuclear translocation of PRMT5 only occurs in the S phase. We co-stained PRMT5 with the S-G_2_ marker cyclin A and quantified the PRMT5 nuclear/cytoplasm ratio in cells within different cell cycle phases. Regardless of cyclin A status, PRMT5 staining showed homogeneous cytoplasmic distribution, and the nuclear/cytoplasmic ratio was similar in both cyclin A^−^ and cyclin A^+^ groups, suggesting that the nuclear translocation of PRMT5 is independent of cell cycle status (fig. S4B).

Given that PRMT5 is responsible for most cellular symmetric dimethylarginine (SDMA) modifications (*34*, *35*), we wanted to quantify SDMA staining pattern in response to RS. Along with HU-induced PRMT5 nuclear translocation, we noted a prominent increase in nuclear SDMA signal. When PRMT5 was inhibited, the HU-induced SDMA signal was greatly diminished (Fig. 4C). Quantification of SDMA nuclear intensity reflected the increase in SDMA after HU exposure in a PRMT5-dependent manner (Fig. 4D). Moreover, Western blot detection of SDMA revealed increases in SDMA signals at multiple molecular sizes upon HU exposure. These increases were abrogated with GSK591 treatment. As PRMT5 protein expression was similar across all treatments, this suggests that HU-induced SDMA was likely due to an augmentation of PRMT5 methylation activity or a redirection of PRMT5 activity toward nuclear targets (Fig. 4E).

As expected, small interfering RNA (siRNA)–mediated PRMT5 knockdown diminished HU-induced SDMA, as observed by immunofluorescence (fig. S4C) and Western blotting (fig. S4D). This phenomenon was also observed in the cervical cancer cell line HeLa (fig. S4E), indicating that this was not cell type specific. Symmetrically dimethylated Sm proteins recognized by the Y12 antibody showed a similar up-regulation pattern in HU in a PRMT5-dependent manner (fig. S4F). Together, our data propose a model in which nuclear translocation of PRMT5 upon RS increases symmetric dimethylation of its nuclear targets, thus modulating the RS response.

### HU treatment leads to increased symmetric dimethylation of ZNF326

**Fig. 5.**
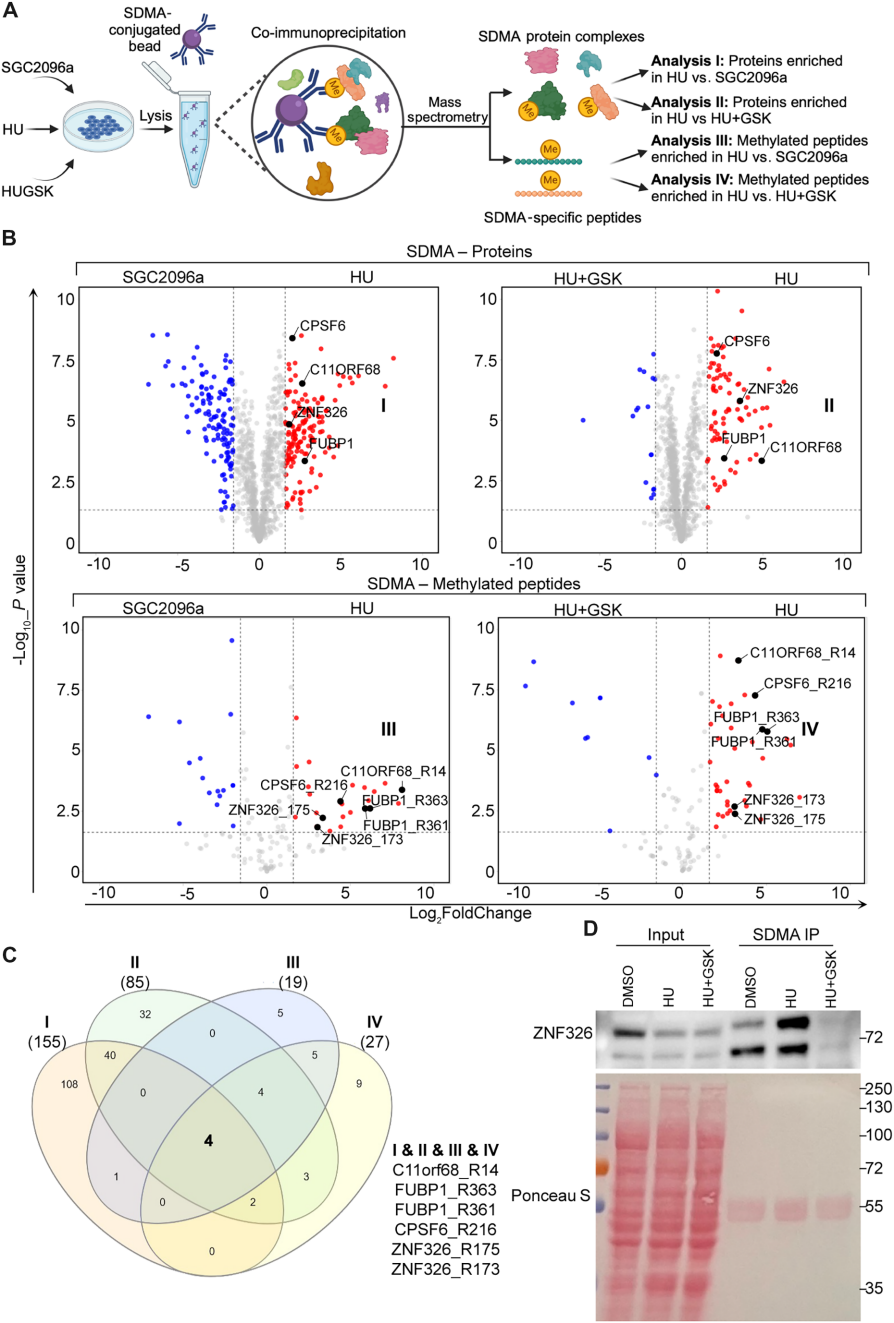
HU treatment leads to increased symmetric dimethylation of ZNF326. (**A**) Schematic workflow to identify symmetric dimethylated (SDMA) proteins and peptides. The illustration was created with BioRender.com. (**B**) Volcano plots displaying SDMA-enriched proteins in MCF10A cells treated with 1 mM HU versus 1 μM SGC2096a, a negative control for PRMT5 inhibitor GSK591, for 6 days (analysis I); SDMA-enriched proteins in MCF10A cells treated with 1 mM HU versus 1 mM HU + 1 μM GSK591 for 6 days (analysis II); SDMA-enriched peptides in MCF10A cells treated with 1 mM HU versus 1 μM SGC2096a for 6 days (analysis III); and SDMA-enriched peptides in MCF10A cells treated with 1 mM HU versus 1 mM HU + 1 μM GSK591 for 6 days (analysis IV). All volcano plots were created with VolcaNoseR app (*72*). (**C**) Venn diagram showing common hits in analysis I, II, III, and IV. The Venn diagram was created with InteractiVenn web-tool (*73*). (**D**) Western blot of MCF10A cells treated with DMSO, HU (1 mM), GSK591(1 μM), or HU (1 mM) + GSK591 (1 μM) for 6 days and co-IP performed with SDMA antibody.

We next aimed to identify regulators of the PRMT5-dependent ISG up-regulation after RS. We hypothesized that the HU-associated up-regulation of ISGs would involve SDMA of one or more protein targets. Upon HU exposure, such elevated SDMA signals would be expected to decrease with concomitant GSK591 exposure. To identify these factors, we performed a quantitative MS experiment. The experimental workflow is detailed in Fig. 5A. We did a pairwise comparison for the relative enrichment of SDMA immunoprecipitated proteins and peptides in HU-treated samples compared to SGC2096a (a negative control compound for GSK591, analysis I) or HU + GSK591 (analysis II) treatments (Fig. 5B and table S2). The comparison of proteins immunoprecipitated by SDMA antibody after HU compared to SGC2096a treatment identified candidates that had elevated SDMA levels or interacted with an SDMA-modified protein upon HU treatment (analysis I). Since we enriched intact proteins and associated factors, we further interrogated our MS data specifically for SDMA-modified peptides for both the HU versus SGC2096a (analysis III) and HU versus HU + GSK591 (analysis IV) comparison (Fig. 5B and table S2). Across these comparisons, we identified C11ORF68, FUBP1, CPSF6, and ZNF326 as candidates that were robustly enriched in HU-treated samples and for which we identified concomitant PRMT5-dependent SDMA modifications on at least one arginine residue (Fig. 5C). We decided to focus on ZNF326 since it is an RNA polymerase II–associated elongation factor (*25*, *36*), possibly affecting inducible genes (i.e., ISGs). We validated the MS results for ZNF326 by immunoprecipitation (IP) with an SDMA antibody and subsequent immunoblotting for ZNF326. In agreement with MS results, ZNF326 was enriched in SDMA IP upon HU treatment (Fig. 5D).

### ZNF326 interacts with PRMT5 and regulates ISG induction by HU

**Fig. 6.**
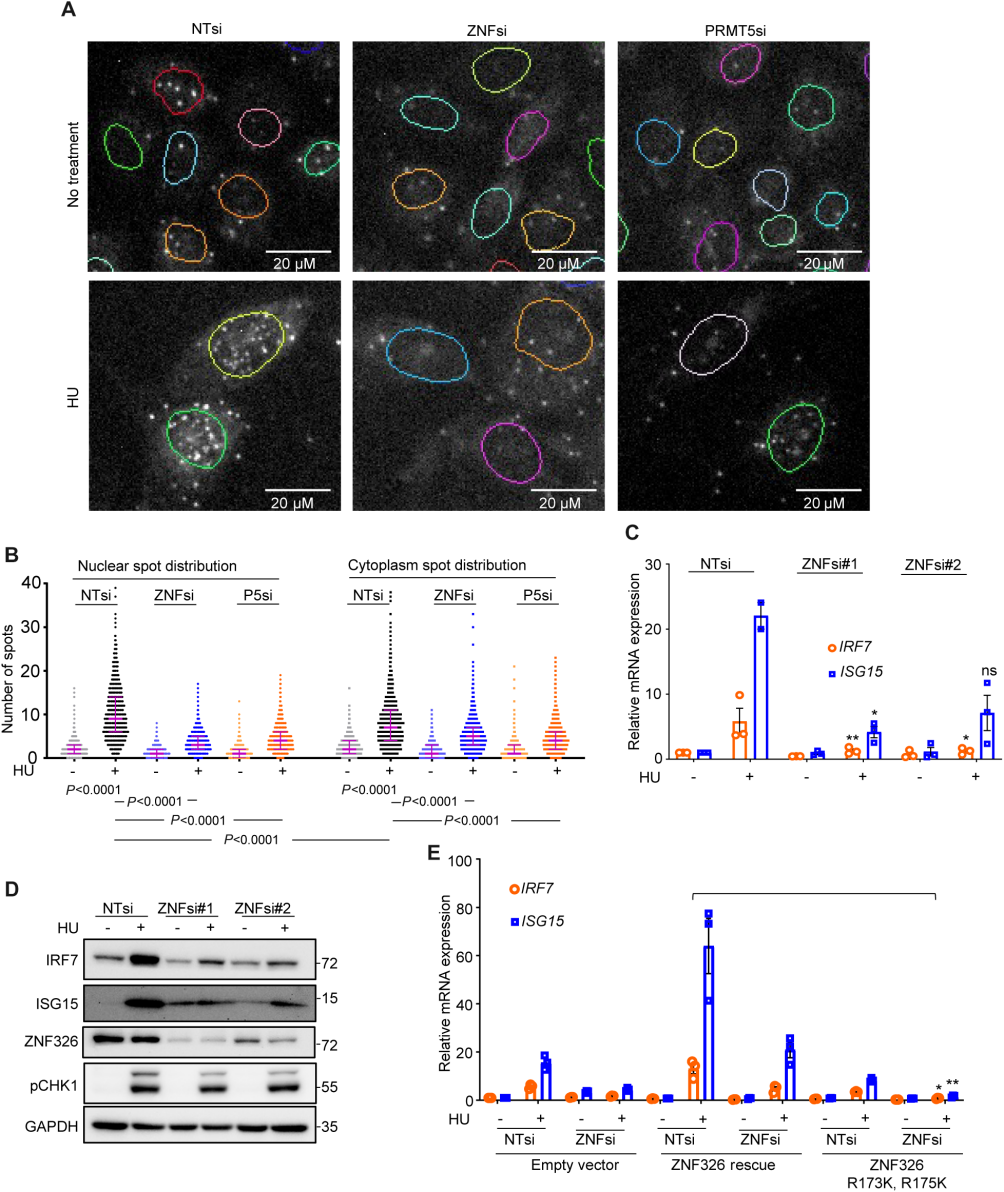
ZNF326 regulates expression of IRF7 and ISG15 induced by HU. (**A**) Control siRNA–, ZNF326 siRNA–, or PRMT5 siRNA–transfected MCF10A cells ± HU (1 mM, 48 hours) subjected to proximity ligation assay (PLA) for PRMT5 and ZNF326. Images were captured and analyzed using the Operetta system. (**B**) A violin plot displaying the median number of spots with interquartile range in nuclear and cytoplasm compartment from at least 1000 cells from the PLA assay as described in (A). Statistical significance for difference in number of spots between treatments was analyzed by unpaired Student’s *t* test of values obtained from a representative of two independent experiments. (**C**) qPCR of MCF10A cells treated with control siRNA– or ZNF326 siRNA–transfected MCF10A cells ± HU (1 mM, 48 hours). Statistical significance was analyzed by unpaired Student’s *t* test of values obtained from three independent experiments. Comparisons were made between ZNF326 siRNAs + HU versus control siRNA + HU. *P ≤ 0.05; **P ≤ 0.01. (**D**) Western blot of MCF10A treated as in (C). Western blot for ZNF326 showed one band, which is different from ZNF326 Western blot with two bands in Fig. 5D. The difference may originate from the usage of IP lysis buffer in Fig. 5D and RIPA in (D). (**E**) qPCR of MCF10A cells with empty vector, WT ZNF326, or ZNF326 with mutation of arginine residues 173 and 175 to lysine constructs transfected with control siRNA or ZNF326 siRNAs ± HU (1 mM). Statistical significance was analyzed by unpaired Student’s *t* test of values obtained from three independent experiments. Comparisons were made between WT ZNF326 transfected with ZNF326 siRNA + HU versus ZNF326 with mutation of arginine residues 173 and 175 to lysine transfected with ZNF326 siRNA + HU. *P ≤ 0.05; **P ≤ 0.01.

To investigate whether PRMT5 directly interacts with ZNF326 to promote symmetric dimethylation after HU treatment, a proximity ligation assay (PLA) was performed. siRNA-mediated knockdown of either ZNF326 or PRMT5 reduced the majority of interaction signals, thereby demonstrating specificity of the ZNF326-PRMT5 PLA and confirming the molecular interaction. HU enhanced ZNF326-PRMT5 interactions in both the cytoplasm and nuclear compartments, with more interactions in the nucleus than in the cytoplasm (Fig. 6, A and B). Finally, to confirm whether ZNF326 is important for HU-induced ISG expression, we knocked down ZNF326 using two different siRNAs targeting ZNF326 and checked for the expression of ISGs. ZNF326 knockdown diminished the increase in HU-induced expression of IRF7 and ISG15 (Fig. 6, C and D). To investigate whether the symmetric dimethylation of ZNF326 at arginine residues 173 and 175 identified by MS analysis is important for the transcriptional regulation of IRF7 and ISG15, we generated MCF10A cell lines expressing ZNF326 siRNA resistant–ZNF326 WT or ZNF326 with arginine to lysine mutation at residues 173 and 175 with comparable ZNF326 expression level (fig. S5). The level of HU-induced ISG expression in the ZNF326 R173K and R175K mutant cell line was reduced when compared to the MCF10A cells expressing WT ZNF326 (Fig. 6E), confirming the importance of PRMT5-mediated methylation of ZNF326 in regulating ISG expression upon HU.

## DISCUSSION

Here, we identified a role for protein arginine methyltransferase PRMT5 in the induction of IFN-stimulated genes (ISGs), specifically in the context of RS. Our observation that PRMT5 promotes HU-induced ISGs and cytokine expression is consistent with a previous report on the positive regulatory role of PRMT5 on intrinsic ISG expression in mesenchymal stromal cells during osteogenic differentiation (*37*) or in a virus-challenged mouse model (*38*). In T cells, PRMT5 has been reported to induce IFN signaling in a graft-versus-host disease mouse model (*39*) and T cell activation (*40*). However, it has been reported that PRMT5 down-regulates cGAS-mediated antiviral immune response (*41*) or type I IFN response in a melanoma mouse model (*42*). The difference of PRMT5 in the regulation of immune response may originate from different models and immune-inducing agents.

SDMA posttranslational modification by PRMT5 regulates diverse cellular processes with direct implications for cancer initiation and development (*32*). Symmetric dimethylation of H2AR3, H4R3 (*43*), and H3R8 (*44*) by PRMT5 is linked to transcriptional repression, whereas SDMA at H3R2 (H3R2me2S) supports H3K4me3 (*45*) and activates FOXP1, which promotes breast cancer stem cell proliferation (*46*). In the cytoplasm, the PRMT5-MEP50-pICln complex methylates Sm proteins, which are critical for the maturation of small nuclear ribonucleoproteins (RNPs), the main components of the spliceosome assembly (*47*–*49*). Therefore, PRMT5 depletion reduces splicing fidelity, resulting in the mis-splicing of many genes, including important genes involved in cell signaling, proliferation, and DDR, such as MDM4 (*50*), ATR (*33*), and TIP60 (*51*). In the nucleus, PRMT5 mediates symmetric dimethylation of ZNF326, a component of the DBIRD elongation complex, to ensure proper exclusion of AT-rich exon (*36*). In the DDR, PRMT5-mediated symmetric dimethylation of FEN1 (*52*), RAD9 (*53*), and TDP1 (*54*) helps maintain genomic integrity upon DNA damage. At DNA double-strand break sites, PRMT5 methylates RUVBL1, which eventually prevents 53BP1 recruitment to the site, thereby promoting homologous recombination repair over the nonhomologous end joining (*55*). In addition to regulating DNA damage repair, PRMT5 prevents RS caused by R-loop accumulation. Symmetric dimethylated DDX5 or RNA polymerase II is required for the recruitment of XRN2 or SETX to resolve R-loops and RS (*56*, *57*). The mechanism by which PRMT5 induces ISG expression had not yet been fully elucidated. In the context of T cells, the increase in IFN expression may be the result of PRMT5 regulation of splicing, which is consistent with the observation that PRMT5 is predominately present in the cytoplasmic compartment, whereby splicing is regulated by PRMT5 (*40*). In a virus-challenged mouse model, PRMT5 was reported to form a complex with cGAS to enhance transcription of ISGs (*38*). However, another study reported that PRMT5 binds and methylates cGAS at Arg^124^ residue, which attenuates its ability to mediate the antiviral immune response (*41*). We did not detect the latter event in our dataset, and our results suggest that a different mechanism is in operation in the context of RS. Upon HU treatment, PRMT5 nuclear translocation was coupled with an increase in nuclear SDMA signals and the PRMT5 nuclear translocation was diminished with the addition of GSK591. Simultaneously, the symmetrical dimethylation level of PRMT5 substrates also decreased (Fig. 4). Therefore, we speculate that HU treatment could promote the formation of a complex between PRMT5 and one/more of its methylated substrate(s). Substrate(s) harboring nuclear localization signal(s) may interact with the PRMT5-related complex to import PRMT5 into the nucleus, where PRMT5 affects RS-induced ISG expression. In line with our hypothesis, SDMA on ZNF326 was substantially enriched after HU in a PRMT5-dependent manner in our MS analysis of immunoprecipitated proteins with SDMA modifications (Fig. 5). The subsequent PLA assay revealed an increase in ZNF326 and PRMT5 expression in the nuclear compartment after HU treatment (Fig. 6A). Efforts to characterize the human interactome of chromatin-associated RNP particles have identified a complex, DBIRD, comprising ZNF326 and DBC1 proteins (*25*). This complex binds RNA polymerase II, exons within AT-rich regions of the mRNA, and influences its propensity to be included in the final transcript, possibly by regulating the elongation rate driven by RNA polymerase II (*25*). Notably, PRMT5 symmetrically dimethylates ZNF326 and loss of PRMT5 mimics ZNF326 loss by promoting exon inclusion within the A+T-rich regions of mRNA (*36*). This is consistent with our overall observation that, upon induction of RS, PRMT5 is translocated to the nucleus and regulates ISG transcription, likely mediated by similar defects in ZNF326-mediated RNA polymerase II elongation.

Increased PRMT5 activity promotes proliferation in certain malignancies (*58*). Therefore, the role of PRMT5 in mediating ISGs in response to RS may have implications in the field of oncology, especially considering the recent clinical trials involving PRMT5 inhibitors (*59*). Given the critical role for PRMT5 to promote ISGs and cytokine transcription in the context of RS, a condition common in cancer, the inhibition of this enzyme may affect the TME. A study reported that deletion of *Prmt5* in mouse T cells leads to the depletion of peripheral CD4^+^ and CD8^+^ T cells, as well as thymic invariant natural killer T cells (*60*). In conclusion, while we describe a role of PRMT5 activity in the context of RS, these findings also have implications for the use of PRMT5 inhibitors concomitantly with RS inducing cancer treatments.

## MATERIALS AND METHODS

### Cell culture

MCF10A cells were cultured in Dulbecco’s modified Eagle’s medium (DMEM)/F12 medium (Invitrogen, #11330-032) supplemented with 5% horse serum (Invitrogen, #16050-122), epidermal growth factor (20 ng/ml) (PeproTech), hydrocortisone (0.5 mg/ml) (Sigma-Aldrich, #H-0888), cholera toxin (100 ng/ml) (Sigma-Aldrich, #C-8052), insulin (10 μg/ml) (Sigma-Aldrich, #I-1882), and 1% penicillin-streptomycin (Invitrogen, #15070-063). HeLa cells were cultured in DMEM (Thermo Fisher Scientific, #11965092) supplemented with 10% fetal bovine serum (FBS; HyClone, #SV30160.03) and 1% penicillin-streptomycin. MKN7 cells were cultured in RPMI medium with 10% FBS (HyClone, #SV30160.03) and 1% penicillin-streptomycin. 

### Drug treatment

For drug treatment without concomitant siRNA addition, 1 × 10^6^ MCF10A cells were seeded in a 10-cm dish. HU (1 mM) or AZD6738 (5 μM) was added for the indicated time points. GSK591 (1 μM) was combined with HU or AZD6738 for the same duration. The medium was replaced with fresh medium and the respective drugs after 3 days for longer duration treatment. The following reagents were used in this study: HU (Sigma-Aldrich, #H8627-5G), AZD6738 (AstraZeneca), GSK591 (Sellekchem, #S8111), dimethyl sulfoxide (DMSO) (Sigma-Aldrich, #8418). EPZ015666 (MedChemExpress, #HY-12727).

### Next-generation RNA-seq

MCF10A cells were treated with HU (1 mM) + DMSO, HU (1 mM) + GSK591 (1 μM), or DMSO alone. RNA was extracted using the RNeasy Mini Kit (Qiagen, #74106) and subjected to deoxyribonuclease (DNase) I treatment (Qiagen, #79254), according to the manufacturer’s protocol. RNA concentration was quantified using a NanoDrop 1000 spectrophotometer. The library preparation and RNA-seq service were provided by Novogene AIT. RNA-seq dataset was deposited onto Gene Expression Omnibus repository with the identifier GSE236930.

### RNA-seq data analysis

FASTQ reads (150–base pair paired-end) were aligned to the human genome GRCh38 primary assembly sequence with STAR (v2.7.5b) (*61*), using default parameters and the Ensembl GRCh38 v99 transcriptome annotation (*62*). Gene and transposable elements (TE)–level expression were quantified using TEcount (v2.2.1) (*63*). Differential gene and TE expression were calculated using DESeq2 (v1.3.0) (*64*) in an R 4.0.3 environment; *P* values were adjusted using the Benjamini-Hochberg method. GSEA was generated from the differential expression signatures using fgsea (v1.16.0) (*65*). Alternative splicing analysis was run using rMATS (v4.1.0) (*66*).

### Quantitative PCR

Cells were lysed and RNA was collected using the RNeasy Mini Kit (Qiagen, #74106) according to the manufacturer’s protocol. RNA concentration was quantified using NanoDrop 1000. RNA (1 μg) was converted to complementary DNA (cDNA) using iScript Reverse Transcription Supermix (Bio-Rad, #1708840) according to the manufacturer’s protocol. cDNA was diluted 1:5. Each quantitative PCR (qPCR) reaction contains 1 μl of diluted cDNA, 0.6 μl of forward-reverse primer mix (10 μM each primer), 7.5 μl of qPCR SYBER Green Master Mix (Primer Design, #Z-PFAST-LR-SY), and 5.9 μl of water. Reactions were performed in a qPCR-compatible 96-well plate and ran on a QuantStudio 3 Applied Biosystems thermocycler with the following conditions: hold stage at 95°C for 20 s, followed by 40 cycles of 1 s at 95°C, 20 s at 60°C, 1 s at 95°C. The melting curve stage was performed at 60°C for 2 s followed by 95°C for 1 s. QuantStudio Design & Analysis Software v1.4.1 was used to process qPCR data. Primer sequences are listed in table S3.

### HiBiT-CXCL10 knock-in cell line generation

We used the Alt-R CRISPR HDR Design Tool to design homology-directed repair (HDR) donor oligos and guide RNAs (gRNAs) targeting the genes of interest. Donor oligos and gRNA sequences are listed in table S3. The CRISPR RNA was mixed with tracrRNA (IDT, #1075927) to make gRNA complex at 50 μM final concentration. The gRNA complex was combined with Cas9 nuclease (IDT, #1081058) to form an RNP complex at final concentrations of 2 μM Cas9 nuclease and 2.4 μM gRNA. MCF10A cells (1 × 10^5^) were resuspended in Opti-MEM (Thermo Fisher Scientific, #31985070) and mixed with the RNP complex and HDR donor oligo in a total volume of 100 μl. The mixture was transferred to 0.2-cm gap electroporation cuvettes (Bio-Rad, #1652086) and electroporated using a Nepagene21 electroporator. After electroporation, MCF10A cells were transferred to culture medium containing 1 μM HDR Enhancer V2 (IDT, #10007910) for 48 hours. Single-cell clones were subsequently obtained by limiting dilution. Genomic DNA was extracted from prospective clones and subjected to PCR using detection primers. Detection primers were listed in table S3. PCR products were sequenced to confirm the desired editing.

### Epigenetic inhibitor screen

MCF10A HiBiT-CXCL10 knock-in cells (2000) were seeded in 50-μl media in each well of 96-well white plate (SPL, #30196) overnight. The day after, DMSO or epigenetic inhibitors were added at a final concentration of 1 μM for 3 days. On day 4, 1 mM HU was added to HU-treated wells for 24 hours, whereas no HU was added to DMSO/epigenetic inhibitor only for control. After incubation time, 50 μl of supernatant was withdrawn from each well and transferred to a new 96-well white plate. The supernatant was mixed with HiBiT extracellular detection at 1:1 ratio according to the manufacturer’s protocol (Promega, #N2420). The remaining cells in 50-μl culture medium were subjected to CellTiter-Glo (CTG) detection for viability (Promega, #G7572). Luminescence readout was obtained with a Tecan Spark plate reader.

### siRNA transfection

The siRNAs used in this study were listed in table S3. The transfection complex containing siRNA and Lipofectamine RNAiMAX Reagent (Thermo Fisher Scientific, #13778-075) were mixed with 5 × 10^5^ MCF10A cells and seeded to a six-well plate at a final siRNA concentration of 25 pmol.

### Western blotting

Following procedures previously described in (*67*), we lysed cells with Pierce radioimmunoprecipitation assay (RIPA) buffer (Thermo Fisher Scientific, #89901) supplemented with Halt Protease and Phosphatase Inhibitor (Thermo Fisher Scientific, #78442) on ice for 30 min. The samples were quantified using a Pierce Bicinchoninic Acid (BCA) assay (Thermo Fisher Scientific, #23225). Samples of at least 20 μg of protein were run on tris-glycine polyacrylamide gel electrophoresis (PAGE) gel. Proteins from the gel were transferred to polyvinylidene difluoride membrane (Bio-Rad, #1620177) overnight at 150 mA at 4°C. Membranes were blocked for at least 30 min in 5% bovine serum albumin (BSA)/tris-buffered saline with 0.1% Tween (TBST) and incubated with the primary antibody (1% BSA in TBST) overnight. The following day, membranes were incubated with either anti-mouse (GE, #NXA931V) or anti-rabbit (GE, #NA9340V) secondary antibodies for 1 hour at room temperature (RT). Chemiluminescent reagents (Millipore, #WBKLS0500) were applied to the membrane, and Western blot images were developed using a film developer (Konica Minolta Film Processor) using a film (Konica Minolta, #A9KN) or a digital ChemiDoc imaging system (Bio-Rad, #12003153). Antibodies used in Western blotting were listed in table S3.

### Immunofluorescence staining

This protocol was used to detect and quantify SDMA, Rad51, γH2AX, and PRMT5 by microscopy. MCF10A cells (5000) were seeded into each well of 96-well clear bottom black plate (Corning, #353219). If siRNA was used in combination with HU, the protocol was outlined in siRNA treatment. Before staining, the cells were washed with phosphate-buffered saline (PBS) and fixed in 4% formaldehyde or 100% ice-cold methanol for 10 min. After washing with PBS, cells were permeabilized with 0.1% Triton X-100 in PBS for 10 min. The cells were again washed with PBS and blocked with 5% BSA in PBS for 30 min. The primary antibodies were diluted to 1:1000 in 5% BSA in PBS. The plates were then incubated at RT for 1 hour. The cells were then washed with PBS containing 1% Tween 20 (PBST) and incubated for approximately 1 hour in the dark with fluorescently conjugated secondary antibodies (1:1000 dilution). After washing the cells again with PBST, equal amounts of PBS were added to each well of a 96-well plate, which was then filled with equal amounts of PBS. The plate was read on a PerkinElmer Operetta following the protocol described in the Operetta manual. The Harmony software (version 4.0) was used for the assay setup and subsequent image analysis. This software enables the specific analysis of the nucleus, cytoplasm, and cell membranes. GraphPad Prism 9 was used to present the data as violin plots. Antibodies used in immunofluorescence staining were listed in table S3.

### Nuclear and cytoplasmic fractionation

Cells were collected by trypsinization and washed once in PBS. Buffer A [10 mM Hepes (pH 7.9), 10 mM KCl, 0.1 mM EDTA, 0.1 mM EGTA, 1 mM dithiothreitol (DTT), 1× Halt Protease and Phosphatase Inhibitor (Thermo Fisher, #78442)] were added to the cell pellets and incubated on ice for 5 min. Pellets were resuspended in buffer A gently using cut yellow tips. NP-40 was added to a final concentration of 0.5% and mixed by vortexing with 5-s high speed followed by 5-s low speed and then put on ice for 1 min and mixed by vortexing 5-s high speed. Using the swing-out rotor centrifuge, we centrifuged the samples at 2000*g* for 5 min at 4°C. The supernatant collected was the cytoplasmic fraction, which was clarified by high-speed centrifuge (15,000*g*, 15 min, 4°C). The remaining nuclear pellets were washed in 500 μl of buffer A without resuspension followed by centrifugation at 2000*g* for 5 min at 4°C in a swing-out rotor centrifuge. The nuclei were extracted using Pierce RIPA buffer (Thermo Fisher Scientific, #89901) supplemented with 1× Halt Protease and Phosphatase Inhibitor (Thermo Fisher Scientific, #78442), 2 mM MgCl_2_, and benzonase (50 U/ml) (Sigma-Aldrich, #E8263) and incubated for 30 min at 4°C. Samples were subjected to sonication at 4°C by the Bioruptor Pico device (Diagenode). The nuclear fraction was clarified by high-speed centrifuge (15,000*g*, 15 min, 4°C). Protein in extracted nuclear and cytoplasmic fractions was quantified using the Pierce BCA Protein Assay Kit - Reducing Agent Compatible (Thermo Fisher Scientific, #23250) according to the manufacturer’s protocol. Samples were run on SDS-PAGE gels and transferred as described for Western blotting.

### Mass spectrometry

MCF10A cells were treated with 1 μM SGC2096a (a negative control for GSK591), 1 mM HU, or 1 mM HU + 1 μM GSK591 for 6 days in biological quadruplicates. The medium was replaced with fresh medium supplied with respective drugs after 3 days. Cells were lysed in IP buffer [10 mM tris (pH 7.5), 150 mM NaCl, 0.5% NP-40, 0.25% sodium deoxycholate, 0.5 mM EDTA (pH 8), 2 mM MgCl_2_, benzonase (50 U/ml) (Sigma-Aldrich, #E8263), 1× Halt Protease and Phosphatase Inhibitor Cocktail (Thermo Fisher Scientific, #78442)] for 30 min at 4°C. Lysates (1.5 mg) were incubated with 20 μl of posttranslational modification (PTM)–conjugated beads (Cell Signaling Technology, #13563). The beads were washed three times in lysis buffer and eluted in 2× NuPAGE lithium dodecyl sulfate (LDS) sample buffer (Thermo Fisher Scientific, #NP0007) with 2.5% β-mercaptoethanol. Sample preparation and MS analysis were performed essentially as previously described (*68*). Briefly, eluted samples were separated on a 12% NuPAGE bis-tris precast gel (Thermo Fisher Scientific) for 10 min at 170 V in 1× Mops buffer, followed by gel fixation using the Colloidal Blue Staining Kit (Thermo Fisher Scientific). For in-gel digestion, samples were destained in destaining buffer (25 mM ammonium bicarbonate; 50% ethanol) and reduced in 10 mM DTT for 1 hour at 56°C followed by alkylation with 55 mM iodoacetamide (Sigma-Aldrich) for 45 min in the dark. Tryptic digest was performed in 50 mM ammonium bicarbonate buffer with 2 μg of trypsin (Promega) at 37°C overnight. Peptides were desalted on StageTips and analyzed by nanoflow liquid chromatography on an EASY-nLC 1200 system coupled to a Q Exactive HF mass spectrometer (Thermo Fisher Scientific). Peptides were separated on a C18 reversed-phase column (25 cm long, 75 μm inner diameter) packed in-house with ReproSil-Pur C18-AQ 1.9 μm resin (Dr. Maisch). The column was mounted on an Easy Flex Nano Source and temperature-controlled by a column oven (Sonation) at 40°C. A 105-min gradient from 2 to 40% acetonitrile in 0.5% formic acid at a flow of 225 nl/min was used. Spray voltage was set to 2.2 kV. The Q Exactive HF was operated with a TOP20 MS/MS spectra acquisition method per MS full scan. MS scans were conducted with 60,000 resolution at a maximum injection time of 20 ms and MS/MS scans with 15,000 resolution at a maximum injection time of 50 ms. Raw files were analyzed with MaxQuant (*69*) version 1.5.2.8 with MaxLFQ quantification (*70*). Carbamidomethylation on cysteines was set as fixed modification, while methionine oxidation, protein N-acetylation, and arginine dimethylation were considered as variable modifications. Search results were filtered with a false discovery rate of 0.01, with the match between runs option activated. LFQ quantitation was based on unique and razor peptides for protein groups with at least two ratio counts. The resulting protein group table was further processed with an in-house script to exclude contaminants (based on the MaxQuant contaminant list), reverse hits, and proteins that were only identified by a modified peptide. Missing values were imputed based on normally distributed values corresponding to the percentage of missing values in each sample for proteins that were quantified in all four biological replicates of at least one treatment condition.

### Cell cycle analysis

MCF10A cells were seeded in a 96-well black plate with a clear flat bottom (Corning, #353219) at 5000 cells per well. Three wells were used for each treatment for 24 hours or 6 days. For 6-day time point, the cells were passaged in between if they became confluent. 5-ethynyl 2´-deoxyuridine (EdU) was detected according to the manufacturer’s protocol (Click Chemistry Tools, #1385). Hoechst staining was used to quantify the DNA content. Subsequent imaging and analysis were performed on a PerkinElmer Operetta, as described in the “Operetta” section. Cell cycle distribution was determined by plotting the log_10_ EdU median intensity against the Hoechst sum intensity.

### IFN ELISA assay

The VeriPlex Human Interferon 9-Plex ELISA Kit was used according to the manufacturer’s protocol (PBL Assay Science, #51500). The plate was imaged using the Azure imaging system. Quantitative analysis of cytokine expression levels was performed using the Q-View software (Quansys Bioscience).

### Proximity ligation assay

After siRNA transfection, 5000 MCF10A cells were seeded into a 96-well black plate with a clear flat bottom (Corning, #353219). After 24 hours, cells were treated with HU for 48 hours before fixation. The PLA for PRMT5 and ZNF326 was performed according to the manufacturer’s protocol (#DUO92101; Sigma-Aldrich).

### ZNF326-expressing cell line generation

DNA for WT ZNF326 or ZNF326 R173K and R175K with modified siRNA recognition sequence was synthesized by Twist Bioscience. DNA was cloned into pMSCVpuro vector (Takara Bio). pMSCVpuro empty vector or with WT ZNF326 or ZNF326 R173K and R175K was cotransfected with retroviral packaging plasmids to human embryonic kidney (HEK) 293T cells. Supernatants from transfected HEK293T cells were collected, filtered through 0.45-μm filter (Sartorius Minisart, #16537), and concentrated using LentiX concentrator (Clontech Laboratories, #631231). MCF10A cells were transduced with concentrated supernatant supplied with polybrene (10 μg/ml) (Sigma-Aldrich, #TR-1003G) for 24 hours and changed to MCF10A culture medium afterward. Puromycin (1 μg/ml) (Gibco, #A1113803) was added 48 hours after transduction to select successfully transduced cells. Lentiviral transduction induced high ISG expression; therefore, ISG expression in response to HU could be detected 2 to 3 weeks after puromycin selection.

### Statistical analysis

The details are provided in the figure legends. Combined biological independents are expressed as the mean ± SEM. Statistical analyses were performed using GraphPad Prism version 9.

## Supplementary Materials

### This PDF file includes:

Figs. S1 to S5 Table S3 Legends for tables S1 and S2 Uncropped Western blots
