# Supplementary material for: A PRMT5-ZNF326 axis mediates innate immune activation upon replication stress
Source: Sci Adv. 2024 Jun 5;10(23):eadm9589. doi: 10.1126/sciadv.adm9589 (PMC11804791; doi:10.1126/sciadv.adm9589)
Supplement: Supplementary file 1 — Figs. S1 to S5 Table S3 Legends for tables S1 and S2 Uncropped Western blots [file sciadv.adm9589_sm.pdf]

Science Advances
AAAS

Supplementary Materials for

A PRMT5-ZNF326 axis mediates innate immune activation upon replication stress

Phuong Mai Hoang *et al.*

Corresponding author: Anand D. Jeyasekharan, csiadj@nus.edu.sg;
Ernesto Guccione, ernesto.guccione@mssm.edu

Sci. Adv. **10**, eadm9589 (2024)
DOI: 10.1126/sciadv.adm9589

The PDF file includes:

Figs. S1 to S5
Table S3
Legends for tables S1 and S2
Uncropped Western blots

Other Supplementary Material for this manuscript includes the following:

Tables S1 and S2

Supplementary figures

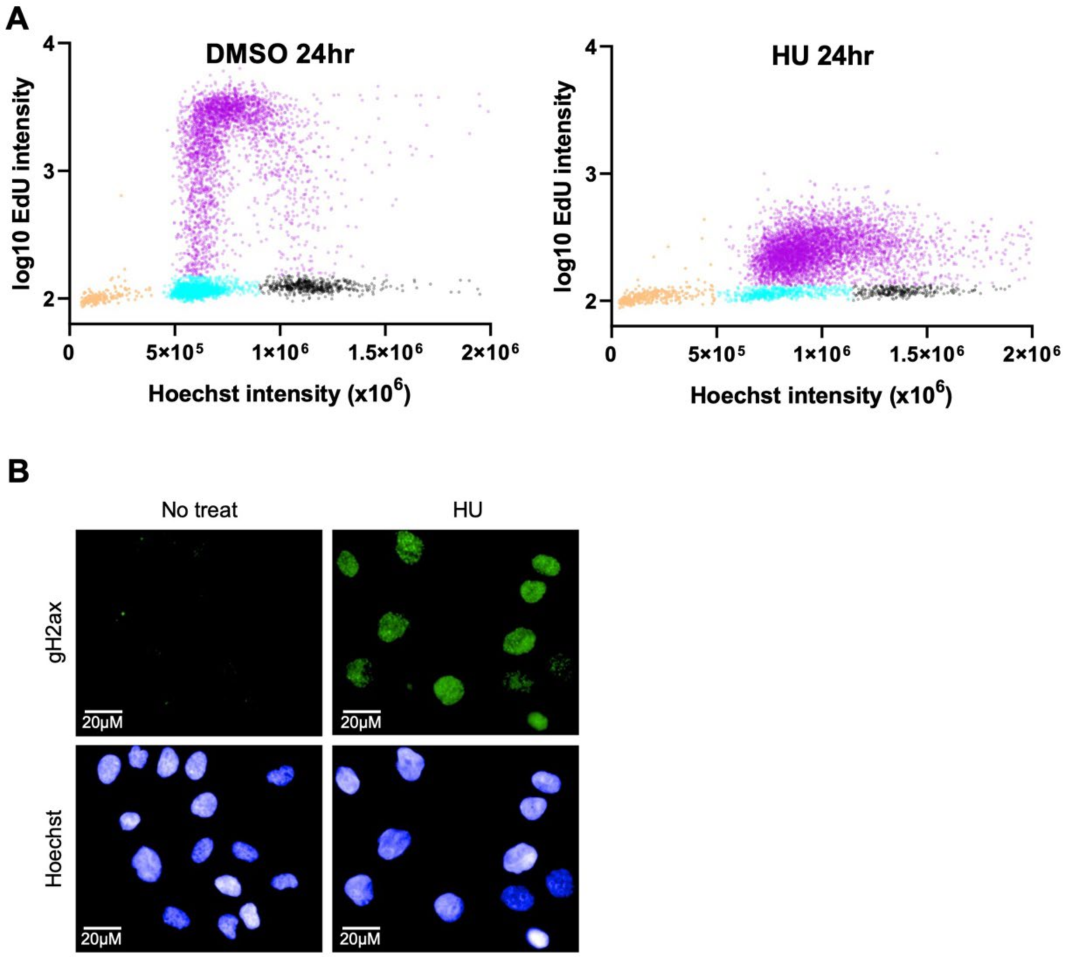

Fig. S1- HU caused cell cycle arrest and DNA damage after 24 hours exposure. A. Cell cycle profile of MCF10A cells treated with DMSO or HU (1mM) for 24 hours. **B.** MCF10A cells treated with DMSO or HU (1mM) for 24 hours and stained for gH2Ax and Hoechst. Images were captured using operetta system.

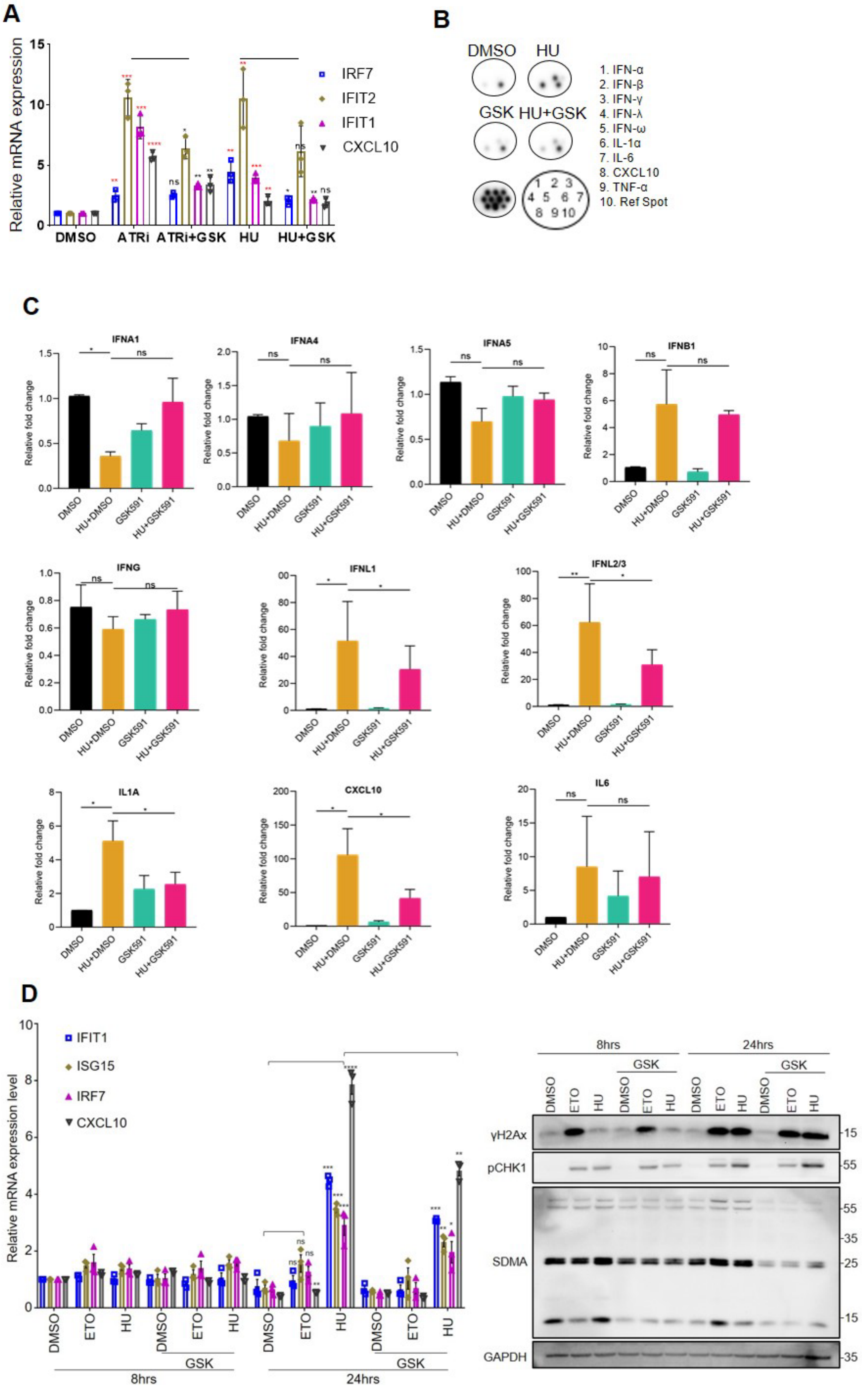

A
Relative mRNA expression
15
10
5
0
IRF7
IFIT2
IFIT1
CXCL10
DMSO
ATRi
ATRi+GSK
HU
HU+GSK
B
DMSO
HU
GSK
HU+GSK
1. IFN-α
2. IFN-β
3. IFN-γ
4. IFN-λ
5. IFN-ω
6. IL-1α
7. IL-6
8. CXCL10
9. TNF-α
10. Ref Spot
C
IFNA1
IFNA4
IFNA5
IFNB1
IFNG
IFNL1
IFNL2/3
IL1A
CXCL10
IL6
Relative fold change
DMSO
HU+DMSO
GSK591
HU+GSK591
D
Relative mRNA expression level
IFIT1
ISG15
IRF7
CXCL10
DMSO
ETO
HU
GSK
8hrs
24hrs
γH2Ax
pCHK1
SDMA
GAPDH

Fig. S2: RS induces a PRMT5 – dependent interferon stimulated gene expression pattern. A. qPCR of MKN7 gastric cancer cell line treated with DMSO, HU (1mM), HU (1mM)+GSK591 (1μM), ATR inhibitor AZD6738 (ATRi) (5μM) or ATRi (5μM)+GSK591 (1μM) for 4 days. Statistical significance was analyzed by unpaired Student t test of values obtained from 3 independent experiments. *p ≤0.05, **p ≤0.01, *** p ≤ 0.001, *** p ≤ 0.0001, ns= not significant. Red * shown statistical difference between MKN7 treated with DMSO versus ATRi or HU. Black * shown statistical difference between MKN7 treated with ATRi versus ATRi + GSK591 or HU versus HU+GSK591. **B.** Representative image from multiplex elisa for interferons and cytokines from supernatants of MCF10A cells treated with DMSO, HU (1mM), GSK591 (1μM), HU (1mM)+GSK591 (1μM) for 6 days. **C.** qPCR of MCF10A cells treated with DMSO, HU (1mM) GSK591(1μM), HU (1mM)+GSK591 (1μM) for 6 days. Statistical significance was analyzed by unpaired Student t test of values obtained from 3 independent experiment. *p ≤0.05, **p ≤0.01, *** p ≤ 0.001, ns= not significant. **D.** Left- qPCR of MCF10A cells treated with single agent DMSO, Etoposide (ETO) (5μM), HU (1mM) or in combination with GSK591 (1μM) for 8 hours or 24 hours. Statistical significance was analyzed by unpaired Student t test of values obtained from 3 independent experiments. Statistical significance was analyzed by unpaired Student t test of values obtained from 3 independent experiment. *p ≤0.05, **p ≤0.01, *** p ≤ 0.001, **** p ≤ 0.0001 ns= not significant. Right- Western blot of MCF10A cells treated as in (A) and blot for γH2Ax, pCHK1, SDMA and GAPDH.

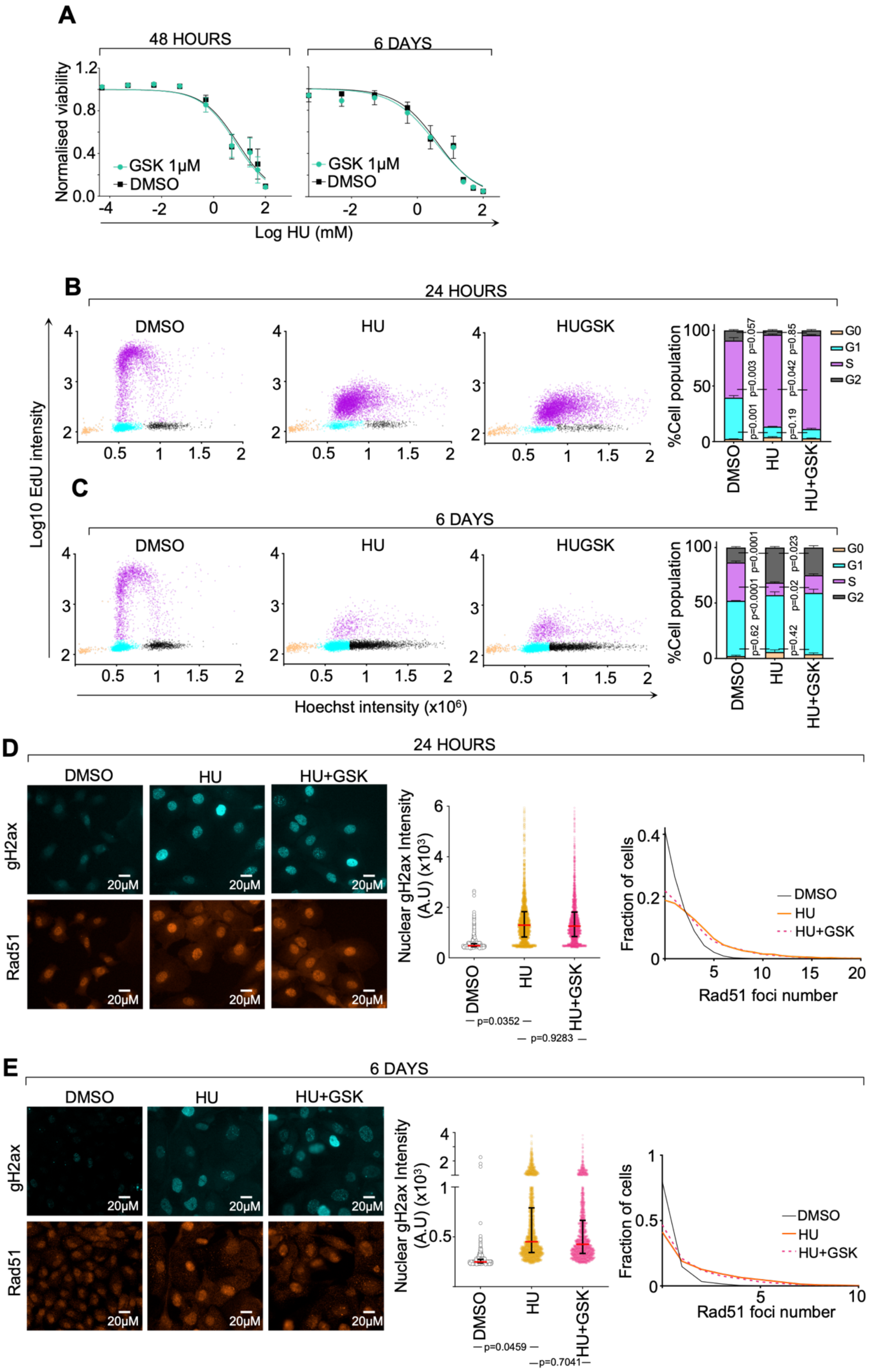
A
48 HOURS
6 DAYS
Normalised viability
GSK 1μM
DMSO
Log HU (mM)
B
24 HOURS
DMSO
HU
HUGSK
%Cell population
G0
G1
S
G2
p=0.057
p=0.003
p=0.001
p=0.85
p=0.042
p=0.19
HU+GSK
C
6 DAYS
Log10 EdU intensity
Hoechst intensity (x10^6)
p=0.0001
p<0.0001
p=0.62
p=0.023
p=0.02
p=0.42
D
24 HOURS
HU+GSK
gH2ax
Rad51
20μM
Nuclear gH2ax Intensity (A.U) (x10^3)
p=0.0352
p=0.9283
Fraction of cells
Rad51 foci number
E
6 DAYS
p=0.0459
p=0.7041

F

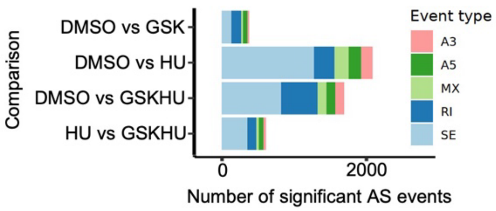

G

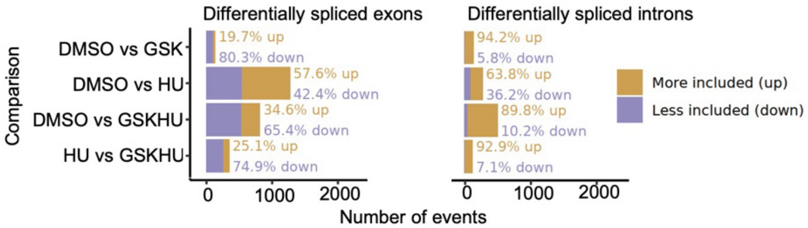

Fig. S3: PRMT5 is dispensable for other HU-induced phenotypes. A. Cell viability of MCF10A treated with different concentration of HU with/ without GSK591 1µM for 48 hours or 6 days, respectively. **B, C.** Left - Cell cycle profile of MCF10A cells treated with DMSO, HU (1mM) or HU (1mM)+GSK591 (1µM) for 24 hours or 6 days, respectively. Right – Bar graphs represent percentage of cell in each phase of cell cycle. p value was analyzed by unpaired Student t test of median values obtained from 3 independent experiments. **D**, **E.** Left - MCF10A cells treated with DMSO, HU (1mM) or HU (1mM)+GSK591 (1µM) for 24 hours (D) or 6 days (E), respectively and stained for gH2Ax and Rad51. Images were captured and analysed using operetta system. Representative images in one of three independent experiment are shown. Middle - Violin plots displaying the nuclear gH2Ax staining intensity of at least 1000 cells for each treatment. p value was analyzed by unpaired Student t test of median values obtained from 3 independent experiments. Right - Histogram displaying Rad51 foci distribution from at least 1000 cells for each treatment. **F.** Pairwise comparison between DMSO, HU (1mM), GSK591 (1µM), HU (1mM)+GSK591 (1µM) for 24 hours in term of number of significant alternative splicing events. **G.** Bar graph to summarise differentially spliced exons (left) and differentially spliced introns (right) significant events with inclusion/ exclusion subtypes in each pairwise treatment comparison.

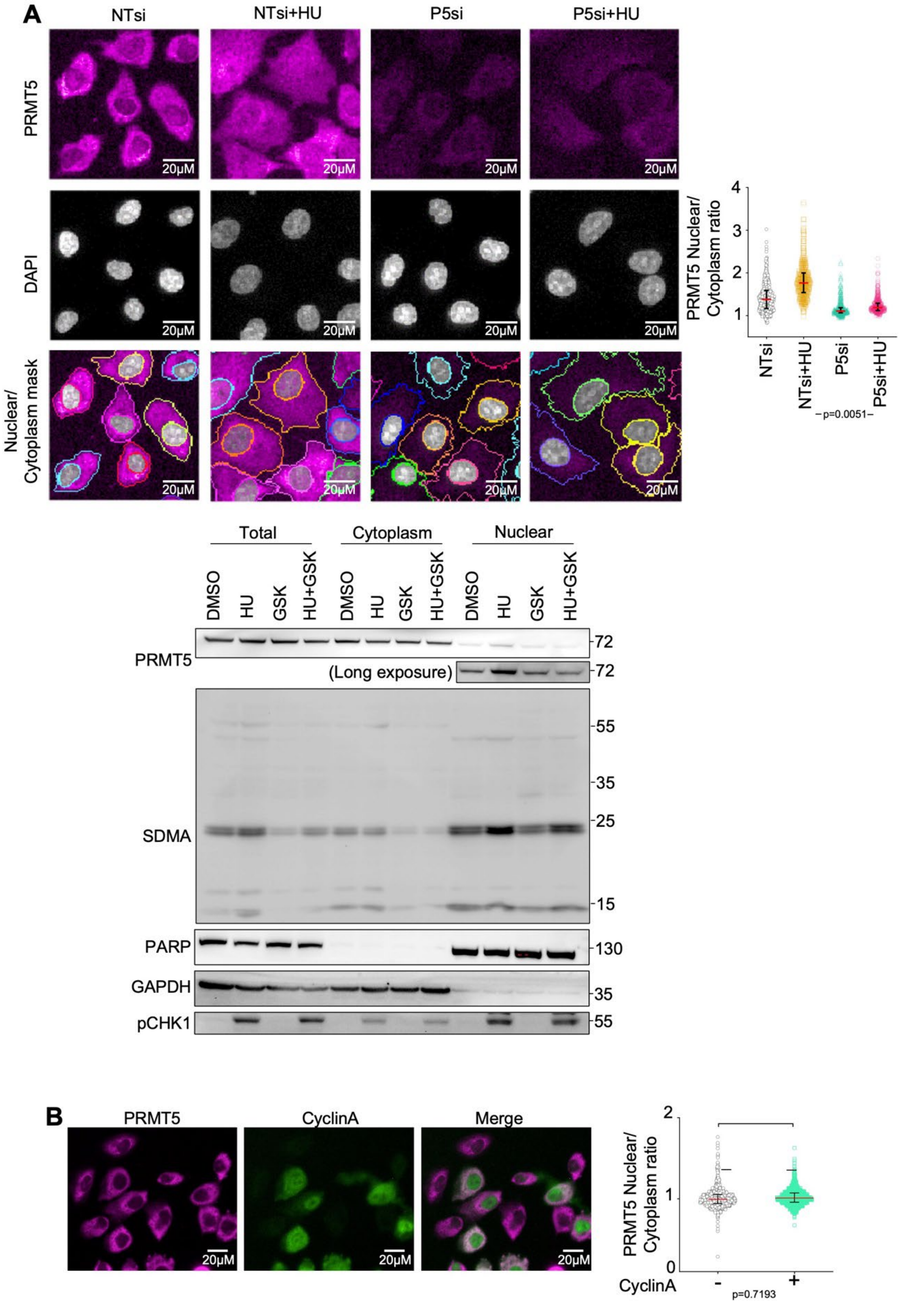

A
NTsi
NTsi+HU
P5si
P5si+HU
PRMT5
DAPI
Nuclear/
Cytoplasm mask
20μM
PRMT5 Nuclear/
Cytoplasm ratio
p=0.0051
Total
Cytoplasm
Nuclear
DMSO
HU
GSK
HU+GSK
PRMT5
(Long exposure)
SDMA
PARP
GAPDH
pCHK1
72
55
35
25
15
130
B
PRMT5
CyclinA
Merge
CyclinA
-
+
p=0.7193

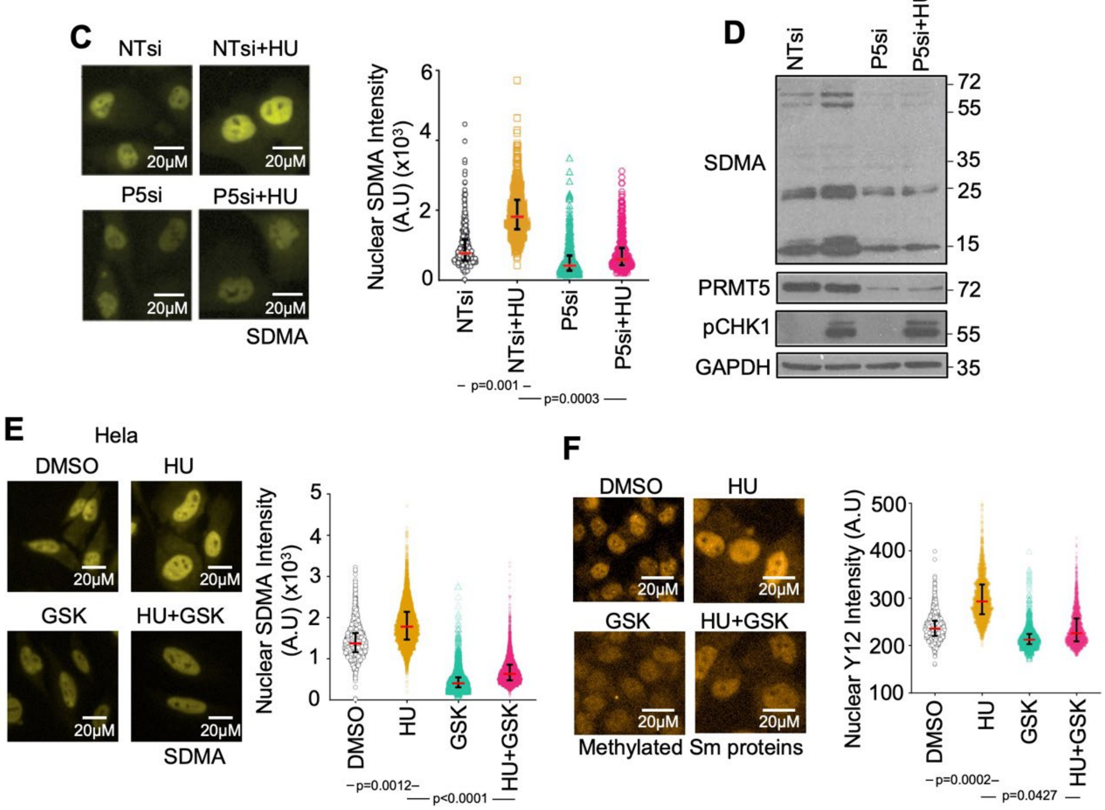
C
NTsi
NTsi+HU
P5si
P5si+HU
20μM
SDMA
Nuclear SDMA Intensity (A.U) (x10³)
6
4
2
0
NTsi
NTsi+HU
P5si
P5si+HU
p=0.001
p=0.0003
D
NTsi
P5si
P5si+HU
SDMA
PRMT5
pCHK1
GAPDH
72
55
35
25
15
72
55
35
E
Hela
DMSO
HU
GSK
HU+GSK
20μM
SDMA
Nuclear SDMA Intensity (A.U) (x10³)
5
4
3
2
1
0
DMSO
HU
GSK
HU+GSK
p=0.0012
p<0.0001
F
DMSO
HU
GSK
HU+GSK
20μM
Methylated Sm proteins
Nuclear Y12 Intensity (A.U)
500
400
300
200
100
DMSO
HU
GSK
HU+GSK
p=0.0002
p=0.0427

Fig. S4: HU leads to an increase in nuclear SDMA. A. Upper- Left-Control siRNA or PRMT5 siRNA transfected MCF10A cells +/- HU (1mM, 24hrs) were stained for PRMT5. Images were captured and analysed using operetta system. DAPI staining represents the nucleus. Nuclear and cytoplasmic boundaries for individual cells are denoted by masks. Representative images in one of three independent experiment are shown. Right- ratio for nuclear PRMT5 staining intensity over cytoplasmic PRMT5 staining intensity from at least 1000 cells treated as in left panel. Lower – Western blot of nuclear and cytoplasmic fractions from MCF10A cells treated with DMSO, HU (1mM, 24hrs), GSK591(1μM, 24hrs), HU (1mM, 24hrs)+GSK591 (1μM, 24hrs). 20μg protein were loaded in each sample with the lysate loading volume ratio of approximately 4 cytoplasm fraction:1 nuclear fraction in equal initial extraction volume. **B.** Left- MCF10A cells +/- HU (1mM, 24hrs) and co-stained for PRMT5 and Cyclin A. Images were captured and analysed using operetta system. Right- A violin plot displaying the ratio for nuclear PRMT5 staining intensity over cytoplasmic PRMT5 staining intensity in cyclin A+ versus cyclin A- population in no treatment group. **C.** Left- Control siRNA or PRMT5 siRNA transfected MCF10A cells +/- HU (1mM, 24hrs) were stained for SDMA. Images were captured and analysed using operetta system. Representative images in one of three independent experiment are shown. Right- A violin plot displaying the nuclear SDMA staining intensity from at least 1000 cells per treatment as in left panel. **D.** Representative western blot for MCF10A cells treated as in (C) from 3 independent experiments. **E.** Left- Hela cells treated with DMSO, HU (1mM, 24hrs), GSK591 (1μM, 24hrs), HU (1mM, 24hrs)+GSK591 (1μM, 24hrs) and stained for SDMA. Images were captured and analysed using operetta system. Right- A violin plot displaying the nuclear SDMA staining intensity from at least 1000 cells per treatment as in left panel. **F.** Left- MCF10A cells treated with DMSO, HU (1mM, 24hrs), GSK591 (1μM, 24hrs), HU (1mM, 24hrs)+GSK591 (1μM, 24hrs) and stained for Y12. Images were captured and analysed using operetta system. Right- A violin plot displaying the nuclear SDMA staining intensity from at least 1000 cells per treatment as in left panel.

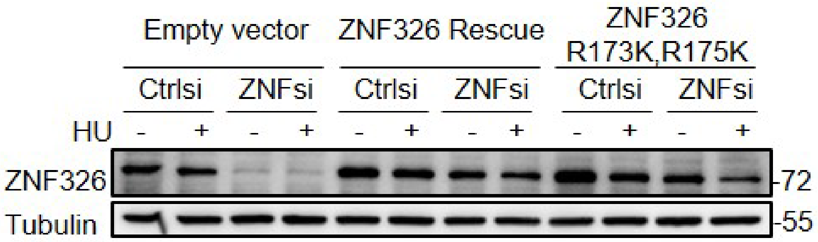

Fig. S5: Generation of MCF10A expressing wild-type ZNF326 or ZNF326 with mutation of arginine residues 173 and 175 to lysine. Western blot of MCF10A cells with empty vector, wild-type ZNF326 or ZNF326 with mutation of arginine residues 173 and 175 to lysine constructs transfected with control siRNA or ZNF326 siRNAs +/- HU (1mM) and blot for ZNF326 and Tubulin.

Table S3. List of materials used in this study

qPCR primers					
Primer	Sequence				
*18S*_R	CCA TCC AAT CGG TAG TAG CG				
*18S*_F	GTA ACC CGT TGA ACC CCA TT				
*CXCL10*_F	CCA CGT GTT GAG ATC ATT GCT				
*CXCL10*_R	TGC ATC GAT TTT GCT CCC CT				
*IFIT1*_F	ACA CCT GAA AGG CCA GAA TG				
*IFIT1*_R	GGT TTT CAG GGT CCA CTT CA				
*IFIT2*_F	GCA CTG CAA CCA TGA GTG AGA				
*IFIT2*_R	AAG GAG TTT TCT CCC TCC ATC AAG				
*IFIT3*_F	CTG GGT GGA AAC CTC TTC AGC				
*IFIT3*_R	TGA CCT CAC TCA TGA TGG CTG TT				
*IFIT2*_F	GCA CTG CAA CCA TGA GTG AGA				
*IFIT2*_R	AAG GAG TTT TCT CCC TCC ATC AAG				
*IFIT3*_F	CTG GGT GGA AAC CTC TTC AGC				
*IFIT3*_R	TGA CCT CAC TCA TGA TGG CTG TT				
*ISG15*_F	CTC TGA GCA TCC TGG TGA GGA A				
*ISG15*_R	AAG GTC AGC CAG AAC AGG TCG T				
*IRF7*_F	CCA CGC TAT ACC ATC TAC CTG G				
*IRF7*_R	GCT GCT ATC CAG GGA AGA CAC A				
**HDR donors**					
Name	Sequence				
HiBiT CXCL10 Donor	ATCCTTGGAAGCACTGCATCGATTTTGCTCCCCTCTGGTTTTA-HiBiT sequence-AGGAGATCTTTTAGACCTGTAAGAAGAGAAAGGGGATATA				
**HDR crRNAs**					
Name	Sequence				
HiBit CXCL10 crRNA	TTTGCTCCCCTCTGGTTTTA				
**HDR Detection primers**					
Primers	Sequence				
CXCL10_HiBit_F	GGTTTTAGCTAATCTTCTTGAACAGCCG				
CXCL10_HiBit_R	GAAGAGATGTCTGAATCCAGAATCGAAG				
**siRNAs**					
Target	Catalog #				
ZNF326#1	s195972				
ZNF326#2	J-019269-05-0002				
PRMT5	sc-41073				
Non-targeting Control Pool	D-001810-10				
**Antibodies**					
Target	Company	Catalog #	Dilution-WB	Dilution-IF	Fixation-IF
Alpha Tubulin	Santacruz	sc-5286	1/1,000		
Cyclin A2	Abcam	ab16726	NA	1/1,000	Methanol
GAPDH	Sigma	G8795	1/10,000		

IRF7	Cell Signaling	13014	1/1,000		
ISG15	Santacruz	sc-166755	1/1,000		
pCHK1 (S345)	Cell Signaling	2348	1/1,000		
phist h2ax (S139)	Merck	05-636	NA	1/1,000	4% PFA
PARP1	Santacruz	sc-74470	1/1,000		
PRMT5	Abcam	ab109451	1/10,000	1/1,000	Methanol
RAD51	Abcam	ab133534	NA	1/1,000	4% PFA
SDMA	Cell Signaling	13222	1/1,000	1/1,000	4% PFA
STAT1 p84/p91	Santacruz	sc-464	1/1,000		
Y12	Professor Joan A. Steitz's laboratory			1/1,000	Methanol
ZNF326	Santacruz	sc-390606	1/1,000		

Other Supplementary Materials for this manuscript include the following:
Table S1: DESeq2 and GSEA analysis for MCF10A +/-HU (1mM) for 24 hours
Table S2: Mass spectrometry analysis for relative enrichment of SDMA immunoprecipitated proteins and peptides.

Uncropped blots for Figure 4E

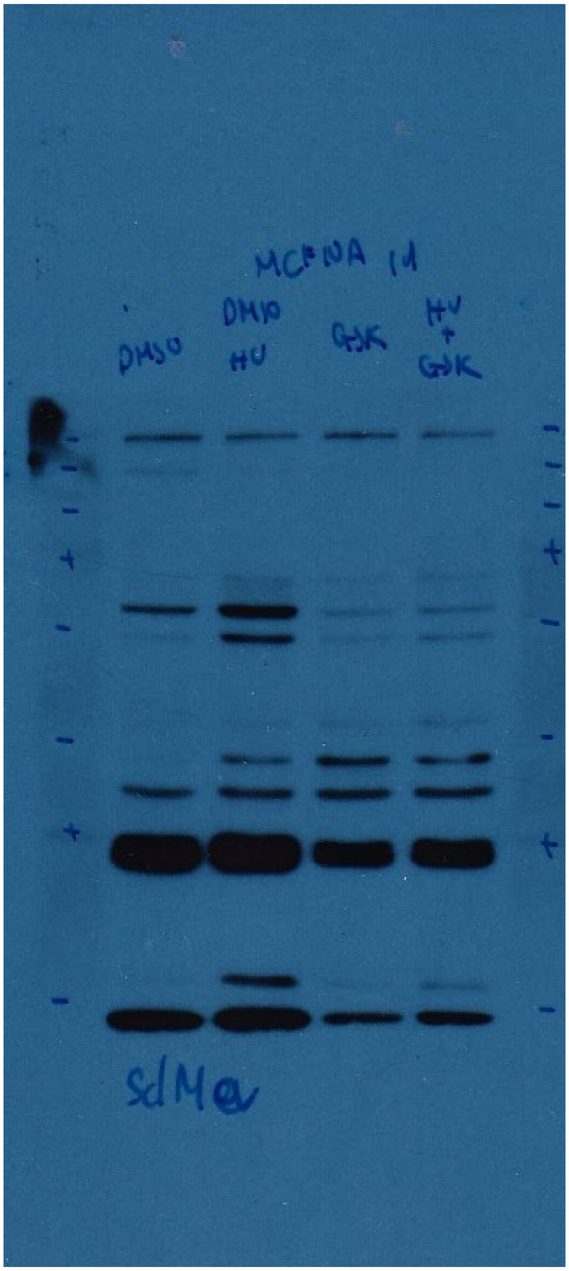

SDMA

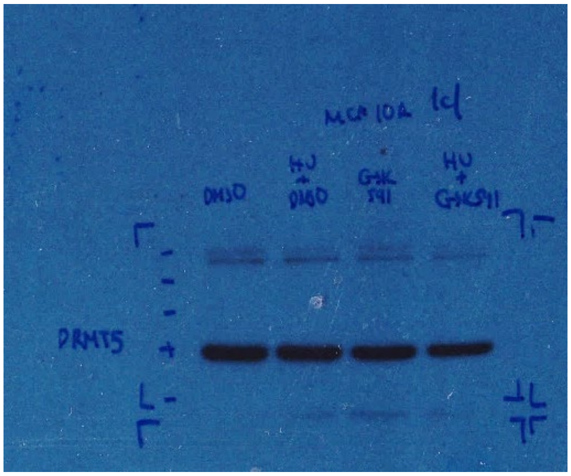

PRMT5

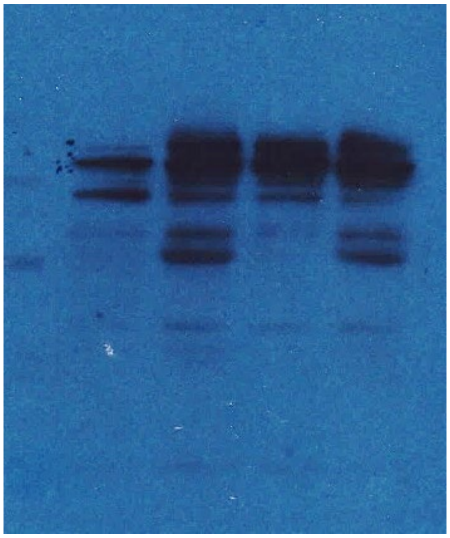
pCHK1

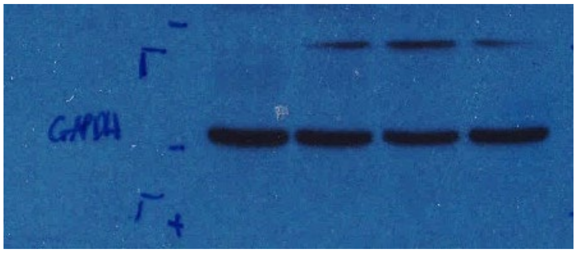

GAPDH
